# The Pampa del Indio project: sustainable vector control and long-term declines in the prevalence and abundance of *Triatoma infestans* infected with *Trypanosoma cruzi* in the Argentine Chaco

**DOI:** 10.1186/s13071-023-05861-7

**Published:** 2023-08-02

**Authors:** Ricardo Esteban Gürtler, Gustavo Fabián Enriquez, María Sol Gaspe, Natalia Paula Macchiaverna, María del Pilar Fernández, Lucía Inés Rodríguez-Planes, Yael Mariana Provecho, Marta Victoria Cardinal

**Affiliations:** 1 Laboratorio de Eco-Epidemiología, Facultad de Ciencias Exactas y Naturales, Universidad de Buenos Aires, Buenos Aires, Argentina; 2 Instituto de Ecología, Genética y Evolución de Buenos Aires (IEGEBA), Consejo Nacional de Investigaciones Científicas y Técnicas (CONICET)-Universidad de Buenos Aires, Buenos Aires, Argentina; 3 Paul G. Allen School for Global Animal Health, Washington State University, Pullman, WA USA; 4 Instituto de Ciencias Polares, Ambiente y Recursos Naturales, Universidad Nacional de Tierra del Fuego, Ushuaia, Argentina; 5 Ministerio de Salud de la Nación, Dirección de Control de Enfermedades Transmitidas por Vectores, Buenos Aires, Argentina

**Keywords:** Gran Chaco, Vector control, Pyrethroid resistance, *Trypanosoma cruzi*, Vector-borne transmission, Sustainability, Disease elimination, Spatial heterogeneity

## Abstract

**Background:**

The Gran Chaco region is a major hotspot of Chagas disease. We implemented a 9-year program aimed at suppressing house infestation with *Triatoma infestans* and stopping vector-borne transmission to creole and indigenous (Qom) residents across Pampa del Indio municipality (Argentine Chaco). The aim of the present study was to assess the intervention effects on parasite-based transmission indices and the spatial distribution of the parasite, and test whether house-level variations in triatomine infection with *Trypanosoma cruzi* declined postintervention and were influenced by household ethnicity, persistent infestation linked to pyrethroid resistance and other determinants of bug infection.

**Methods:**

This longitudinal study assessed house infestation and bug infection with *T. cruzi* before and after spraying houses with pyrethroids and implemented systematic surveillance-and-response measures across four operational areas over the period 2007–2016. Live triatomines were individually examined for infection by optical microscopy or kinetoplast DNA (kDNA)-PCR and declared to be infected with *T. cruzi* when assessed positive by either method.

**Results:**

The prevalence of infection with *T. cruzi* was 19.4% among 6397* T. infestans* examined. Infection ranged widely among the study areas (12.5–26.0%), household ethnicity (15.3–26.9%), bug ecotopes (1.8–27.2%) and developmental stages (5.9–27.6%), and decreased from 24.1% (baseline) to 0.9% (endpoint). Using random-intercept multiple logistic regression, the relative odds of bug infection strongly decreased as the intervention period progressed, and increased with baseline domestic infestation and bug stage and in Qom households. The abundance of infected bugs and the proportion of houses with ≥ 1 infected bug remained depressed postintervention and were more informative of area-wide risk status than the prevalence of bug infection. Global spatial analysis revealed sharp changes in the aggregation of bug infection after the attack phase. Baseline domestic infestation and baseline bug infection strongly predicted the future occurrence of bug infection, as did persistent domestic infestation in the area with multiple pyrethroid-resistant foci. Only 19% of houses had a baseline domestic infestation and 56% had ever had ≥ 1 infected bug.

**Conclusions:**

Persistent bug infection postintervention was closely associated with persistent foci generated by pyrethroid resistance. Postintervention parasite-based indices closely agreed with human serosurveys at the study endpoint, suggesting transmission blockage. The program identified households and population subgroups for targeted interventions and opened new opportunities for risk prioritization and sustainable vector control and disease prevention.

**Graphical abstract:**

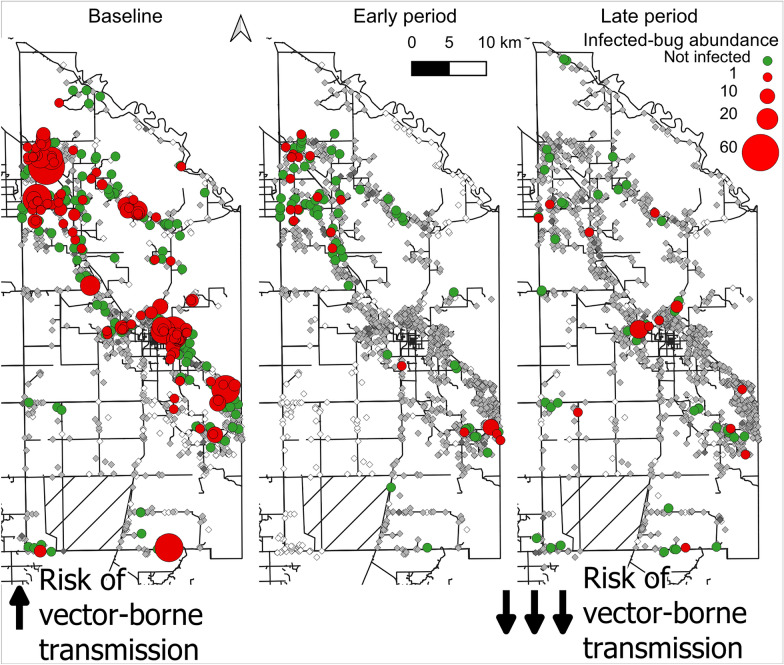

**Supplementary Information:**

The online version contains supplementary material available at 10.1186/s13071-023-05861-7.

## Background

Triatomine bugs are the vectors of the protozoan parasite *Trypanosoma cruzi* (Kinetoplastida, Trypanosomatidae), the etiologic agent of Chagas disease. With 5–7 million people infected with *T. cruzi*, 25–30% of whom are potentially affected by the disease [1], Chagas disease was targeted for elimination as a public health problem in the 2020–2030 roadmap set by WHO [2]. Several regional intergovernmental initiatives coordinated control efforts over the 1990s to interrupt the vector-borne and transfusional transmission of *T. cruzi* [3–5]. Consequently, domestic parasite transmission mediated by *Triatoma infestans* was halted across a large endemic region covering Brazil, Chile, Uruguay, Paraguay and sections of Argentina and Bolivia [6]. *Rhodnius prolixus*, a major vector of Chagas disease, was apparently eliminated from Central America, and control of *Triatoma dimidiata*, also a major vector, progressed substantially [7, 8]. For many triatomine species with extensive sylvatic foci and frequent infection with *T. cruzi*, such as *Panstrongylus megistus* and members of the *Triatoma brasiliensis* complex, the risk of vector-borne transmission to humans may not be negligible and requires an in-depth appraisal.

House spraying with pyrethroid insecticides has been the mainstay of Chagas disease control programs aimed at suppressing domestic triatomines since the mid-1970s. Despite the impressive impact of community-wide insecticide spraying on triatomine numbers during the attack phase, house infestation usually resurges after a variable number of years unless effective surveillance-and-response measures are consistently applied (reviewed in [9]). The process of house reinvasion and re-establishment of domestic triatomine colonies (i.e. reinfestation) is fueled by sources of triatomines in peridomestic or sylvatic habitats and untreated houses, the lower efficacy of pyrethroids in outdoor structures [9], pyrethroid resistance [10, 11] and several socioecological processes [12]. The joint action of these factors hampers the sustainability of long-term vector control efforts, especially in remote, resource-poor communities, leading to heterogeneous patterns of house infestation and parasite transmission. Hence the slow pace of progress in the elimination of *T. infestans* in large sections of the Gran Chaco ecoregion, a global hotspot of neglected tropical diseases (NTDs) and the region perhaps most affected by Chagas disease in the Americas [12–14]. The challenge of eliminating *T. infestans* from the Gran Chaco, given current tools and organizational arrangements, merits research at multiple scales [15].

Chagas disease vector control programs have traditionally measured the effectiveness of interventions via a set of indices representing house infestation and colonization, triatomine density and crowding and natural infection (i.e. percentage of tested triatomines infected with *T. cruzi*) [3, 16–19]. However, most program or trial evaluations have relied on house infestation and density indices, and implicitly or explicitly excluded the use of triatomine infection based on its labor-intensive nature and requirement of laboratory equipment and skilled technicians [17–19], with some notable exceptions (e.g. [20, 21]). Detection of *T. cruzi* has usually been conducted by microscopic examination of wet-mount slides containing a drop of rectal contents diluted with saline solution; positive outcomes are based on the presence of either epimastigotes or trypomastigotes. *Blastocrithidia triatomae,* another member of the Trypanosomatidae and very rarely found in field-collected *T. infestans* [22, 23], is easily differentiated by optical microscopy (OM) because of the occurrence of multiple cysts attached to the flagellum; *Trypanosoma rangeli* does not seem to occur in field-collected *T. infestans*. In a systematic review of papers related to triatomine control interventions published over the period 1948–2009, only a small fraction (23%, 21/93) reported vector infection with *T. cruzi* [24, see Table 5 in ref. 25]. Long-term studies were much fewer. In the dry Argentine Chaco, the prevalence of *T. cruzi*-infected *T. infestans* fell both in domiciles and peridomiciles over the initial 5 years after full-coverage insecticide sprays followed by selective treatments [22] and further declined over the next 12 years [see Supplementary Information Figure 5 in ref. 26]. By contrast, the prevalence of *T. cruzi* infection in *T. infestans* was not associated with time elapsed since the last application of insecticide in selected houses across multiple rural locations in Argentina [27]. Other triatomine species with extensive sylvatic foci displayed heterogeneous patterns of postspraying bug infection [28–30]. Despite being a key component of transmission risk, triatomine infection with *T. cruzi* and its spatiotemporal variations before and after control actions have received little attention in the Chagas disease literature.

The use of separate indices representing house infestation, triatomine abundance and infection at the relevant spatial scales is far from ideal. Both theory and empirical observations support the premise that the incidence of vector-borne human pathogens increases non-linearly with the product of vector-to-host density, the prevalence of vector infection and human-vector contact rates, as encapsulated in indices such as the entomological inoculation rate traditionally used by malaria control programs [31, 32]. For Chagas disease, the relative abundance of *T. cruzi*-infected triatomines per unit effort in domestic habitats (i.e. domicile, intradomicile or human sleeping quarters) is the composite index most closely related to human and companion animal infection with *T. cruzi* [27, 33–40]. In practice, lack of bug infection data and use of disconnected entomological indices limit our understanding of transmission dynamics and assessment of intervention effects.

As a sequel to a research program on the main sources of house reinfestation with triatomines across the Gran Chaco and the Brazilian *cerrado* and *caatinga* ecoregions, the Pampa del Indio project in northeastern Argentina scaled up the operations municipality-wide to test whether intensified, high-quality vector control actions were able to suppress *T. infestans* in a sustained fashion and to interrupt the domestic transmission of *T. cruzi* infection across all rural villages grouped in four (1–4) operational areas [41]. Domestic infestation and triatomine abundance were significantly greater in households of indigenous people (Qom) than in those of Creole people. Domestic and peridomestic infestation were strongly and positively associated at the household level, as was preintervention (baseline) and postintervention house infestation [9, 41]. Community-wide residual spraying with pyrethroids strongly dropped house infestation and relative bug abundance over the first 12 months postintervention (MPI) [42–44]. Incipient-to-high levels of pyrethroid resistance reduced the effectiveness of pyrethroid treatments, mainly in sections of area 1, requiring selective applications of an organophosphate insecticide. House infestation rarely occurred from 60 MPI onwards and fell below 1% at the endpoint [41]. All of the *T. infestans* tested at baseline and most dogs and cats were infected with *T. cruzi* discrete typing units TcVI and TcV in area 1 [45, 46]. *Triatoma sordida,* another abundant species that occasionally invaded domestic premises without establishing viable colonies [47], displayed low [48] or nil [43] infection with *T. cruzi* before and after interventions. The baseline seroprevalence of human infection with *T. cruzi* (range: 29.0–39.8%) was positively related to the domestic abundance of infected *T. infestans* and was greater among Qom households [37, 39]. District-wide serosurveys at the endpoint combined with historical house infestation data supported the conclusion that the vector-borne transmission of *T. cruzi* infection to rural residents had been interrupted [49].

In the study reported here, we integrated house-level data on bug infection, house infestation and the relative abundance of *T. infestans* to build parasite-based transmission indices, examine their degree of correlation and assess the outcomes of control interventions on the space–time distribution of parasite-based indices over 2007–2016. Based on findings described above, we hypothesized that all indices would decline as cumulative surveillance-and-control efforts increased over time. Household ethnicity, baseline domestic infestation and developmental stage were considered a priori to be effect modifiers of the main relation at issue based on well-established empirical evidence (e.g. [22, 38, 50]). We assessed whether the relative odds of future bug infection across the 9 years was associated with: (i) baseline domestic infestation or baseline bug infection; (ii) persistent domestic infestation (i.e. a marker of increased bug survival linked to local pyrethroid resistance in area 1); and (iii) the frequency of occurrence of bug infection (i.e. number of survey occasions in which infection was detected). For current purposes, persistent infestations or foci are defined as those in which *T. infestans* bugs were caught by timed searches at the same house compound in at least two surveys over 0–28 MPI. We hypothesized that, in area 1, the relative odds of bug infection at the household level would not significantly differ before and after the attack phase, and that the prevalence of bug infection would rise with an increasing number of survey occasions in which bug infection was detected, based on the assumptions that persistent foci implied the enhanced survival of triatomines (regardless of their *T. cruzi*-infection status) and host infection and vector contact rates remained stable over the 2-year period (implying no effective host treatment with parasiticidal drugs and no differential migration or death of infected hosts). We frequently focus on area 1 as a study case because of the occurrence of persistent infestation, presence of pyrethroid resistance and an extended time series with numerous observations on bug infection. This paper may be the first to use composite indices to assess the long-term outcomes of district-wide elimination efforts and to investigate the relationship between pyrethroid resistance, vector control failures and triatomine infection in the context of sustained control efforts.

## Methods

### Study area

**Fig. 1:**
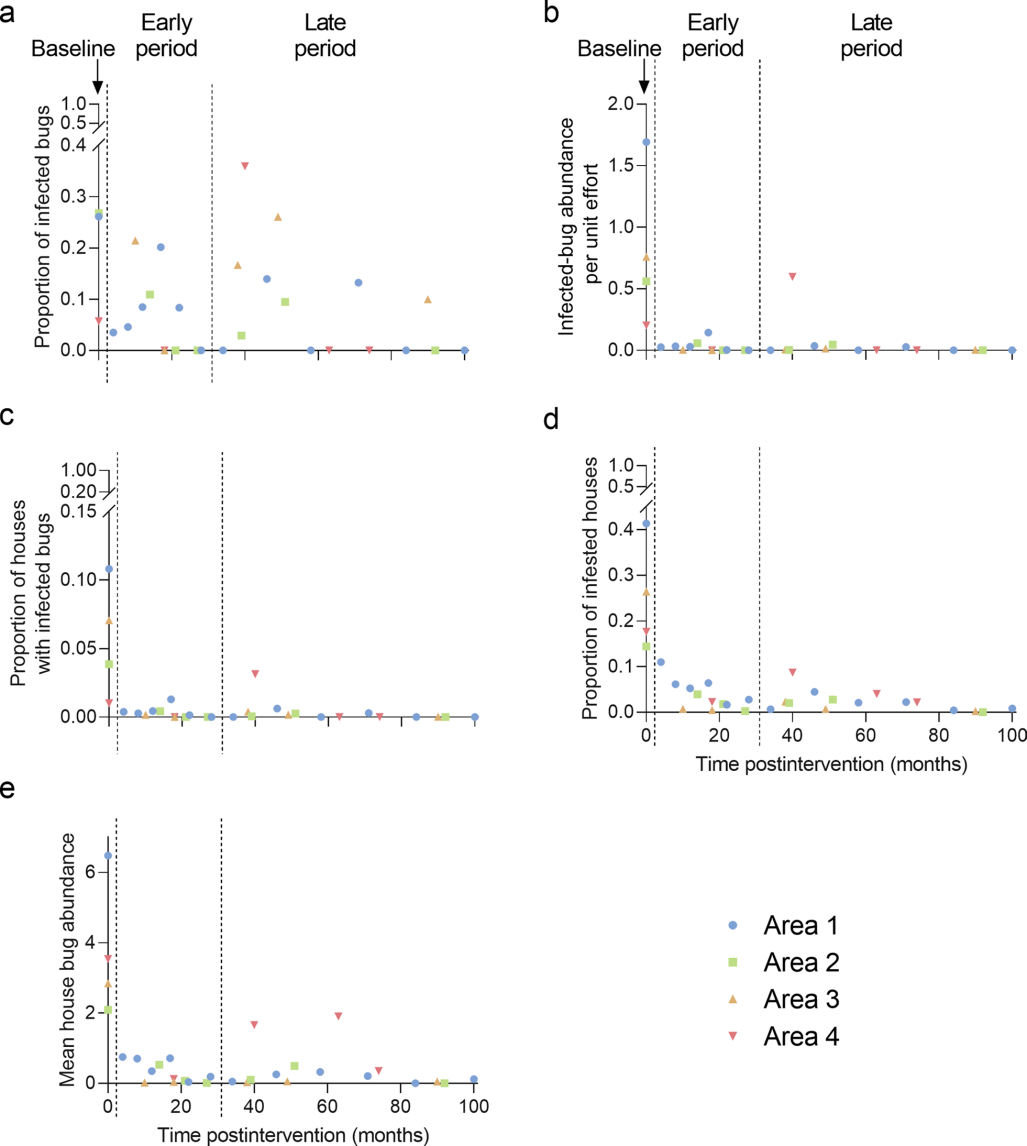
**a**–**c** Scatterplots of the mean prevalence of *Trypanosoma cruzi* infection in *Triatoma infestans* (**a**), abundance of infected bugs per unit effort (**b**) and proportion of houses with at least one infected triatomine (**c**).** d**,** e** House infestation (**d**) and bug abundance per unit effort (**e**) according to time postintervention (in months) for each operational area in Pampa del Indio, 2007–2016. All bug ecotopes included. Data for (**d**) and (**e**) are from [41], with permission

The intervention program was conducted during 2007–2016 in the municipality of Pampa del Indio (Chaco Province, Argentina, located on the interface between the dry and humid Chaco. This municipality (also referred to as the “district”), which was inhabited by roughly similar frequencies of Creole and indigenous (Qom) households and comprised 32 dispersed rural settlements with 1546 housing units at baseline, was divided into four areas (1–4) [41: see Fig. 1]. Both groups differed in several respects and displayed large indices of social deprivation [43, 50]. Qom households were heavily aggregated in areas 2 and 3 and in a few other villages.

Houses generally had mud walls and thatched or corrugated tarred cardboard roofing at baseline [44, 51, 52]. House compounds consisted of a domicile or domestic habitat (i.e. a separate structure used as human sleeping quarters), and a peridomestic habitat including a patio and other near-by outbuildings such as kitchens, storerooms and chicken coops. The last campaign of house spraying with insecticide had been conducted over 1996–1999, with the exception of a few selective insecticide treatments conducted by local health personnel in area 3.

### Study design

The before-after intervention program was designed to assess the effects of a conventional area-wide spraying with pyrethroid insecticide (attack phase), followed by systematic vector surveillance-and-response measures, on house infestation with and abundance of *T. infestans* in the context of persistently infested neighboring municipalities. Ethical considerations precluded the use of a control (no intervention) area. The program aimed at achieving full coverage of all rural housing units registered at baseline (*n* = 1546). Throughout this article, “intervention” refers to the initial area-wide insecticide spraying in each operational area. Postintervention surveys were conducted at defined times after the baseline assessment of house infestation, which is used as time zero of the intervention program (0 MPI).

The intervention program was replicated asynchronically in the four areas, as described elsewhere [41: Table S1]; the dates for interventions and data collection are given in Additional file 1: Figure S1. The protocol included: (i) a district-wide exploratory survey for geographical reconnaissance and collection of preliminary data on house infestation, bug infection and sociodemographic characteristics in September 2007; (ii) an area-wide baseline survey of house infestation with triatomines using timed-manual searches assisted with 0.2% tetramethrin (Espacial, Buenos Aires, Argentina) combined with a household survey of sociodemographic and environmental features; (iii) an area-wide house spraying of pyrethroid insecticide (suspension concentrate deltamethrin at 25 mg/m^2^ [K-Othrin; Bayer AG, Leverkusen, Germany] or beta-cypermethrin at 50 mg/m^2^ [Sipertrin; Chemotecnica S.A., Ezeiza, Argentina]) using standard procedures, which covered 92.7% of all registered houses; (iv) scaling up of program activities to the four areas over 2007–2010; and (v) the surveillance phase, which comprised the periodic evaluation of house infestation and sociodemographic features immediately followed by selective spraying of detected foci with a pyrethroid insecticide (over 2008–2016). With very few exceptions, all houses found to be infested with *T. infestans* in the timed searches were immediately sprayed with insecticide. For the purposes of the present study, the surveillance phase was divided into an early intervention period (for consolidation) spanning 4–28 MPI (when malathion was used to extinguish the persistent foci) and a late intervention period spanning 34–100 MPI. Seventeen house compounds that showed a persistent house infestation with *T. infestans* were treated with a standard dose of malathion (Onix; Cheminova, Lemvig, Denmark, at 1 g/m^2^) mostly over 21–28 MPI (including 2 repeat treatments). The program implemented 1823 insecticide sprays across the 9-year period, 82.3% of which occurred during the attack phase [41].

The periodicity of house infestation surveys differed between areas as part of an adaptive management strategy in response to survey outcomes within resource constraints; area 1 was selected for detailed research of the spatiotemporal dynamics of house infestation. A district-wide survey aiming at full coverage was conducted in April–May 2016 to assess endpoint house infestation. As part of a parallel insecticide trial, 220 housing units located in adjacent districts to the south of Pampa del Indio were sprayed with pyrethroid across late 2014 to 2015.

Households were assigned to the Qom ethnic group based on whether the residents spoke the Qom language and participated in traditional Qom organizations, and also took into account the tenants’ physical features and cultural practices. The few households (< 5%) with at least one person self-identified as Qom were classified as Qom households. Ad-hoc consultation with local healthcare personnel, community leaders and other referents were made to fill in the missing data for household ethnicity.

Etiological treatment of *T. cruzi*-seropositive children aged ≤ 18 years with benznidazole or nifurtimox was conducted in area 1 during September 2010–March 2011 [53], and was subsequently scaled up to include residents aged ≤ 21 years and women in child-bearing age [49].

### Vector surveys

Each housing unit was identified with a numbered plaque and georeferenced with a GPS receiver (Trimble GeoXM [Trimble Inc., Westminster, CO, USA] or Garmin Legend [Garmin Ltd., Olathe, KS, USA]) at baseline. Maps used base layers from the Instituto Geográfico Nacional (Argentina) (https://www.ign.gob.ar/NuestrasActividades/InformacionGeoespacial/CapasSIG). The maps were created in QGIS version 3.28.3 geographic information system platform based on the data collected within the scope of this study.

At each survey, all housing units were classified by their current state (occupied or vacant) and whether they ceased to exist (demolished) or were new. We assessed house infestation with triatomines through: (i) timed manual searches assisted with an aerosol to dislodge the insects from their refuges (0.2% tetramethrin); (ii) householder collection of any triatomine into the plastic bags we had provided for this purpose; and (iii) insecticide spray-related collections of triatomines spotted during or after house spraying, as described elsewhere [41]. In some infested houses, the stipulated search time was extended to increase sample sizes for assessment of bug infection and other purposes (i.e. post-timed searches). Search teams included two or three skilled bug collectors and one supervisor. Each domestic and peridomestic site was inspected for triatomines by one person during approximately 15 min. House-level infestation with *T. infestans* is defined by the finding of at least one live bug (except for eggs) by timed searches in any habitat within the house compound. Relative bug abundance was calculated as the total number of live *T. infestans* caught in the timed manual searches per unit effort across all inspected habitats at the house-compound level. House infestation and triatomine abundance were computed for both occupied and vacant housing units inspected for infestation. Ecotope or habitat refers to a category of individual places surveyed for triatomines (e.g. chicken coop, goat corral); “sites” are habitat units, such as a particular chicken coop.

All bugs collected in a site were first stored separately in a labeled plastic bag. They were then transported to the field laboratory where they were identified taxonomically and counted according to species, developmental stage and sex; examined individually for *T. cruzi* infection (as described in section Triatomine infection); placed in a microtube; and finally assigned a unique identifier code by insect. The offspring of 76 T. *infestans* populations collected across areas and intervention periods were tested for pyrethroid resistance using discriminating dose assays as previously described [43]. The distribution of resistance status was classified as susceptible (33%), incipient (22%), moderate (28%) and high (17%) resistance [41]. Other pieces of evidence also indicated vector control failures caused by pyrethroid resistance, including failure to suppress a heavily infested, large peridomestic structure covered with cloth netting despite repeated pyrethroid applications [54]; the stage structure of triatomine populations collected in persistent foci slightly after the attack phase; and spatial clustering of foci that required multiple insecticide treatments [41].

### Triatomine infection

For individual detection of *T. cruzi* infection by OM, we restricted examination to live third-instar nymphs and later stages. The mean elapsed time between bug collection and OM-based diagnosis was 21 (standard error [SE]: 0.2) days among 5346 bugs examined for infection, compared to 13 (SE: 0.4) days in area 2, 18 (SE: 0.4) days in area 4, 18 (SE: 0.2) days in area 1 and 37 (SE: 0.5) days in area 3. This lag allowed some triatomines to molt, especially among the early-instar nymphs during spring–summer, and to develop an established, detectable infection while kept under laboratory conditions for 1–3 weeks. For individual OM-based diagnosis, a drop of feces obtained by abdominal compression was diluted with saline solution on a slide, covered with a 22 × 22-mm coverslip and examined thoroughly for motile trypanosomes at 400× magnification under a binocular microscope. Forceps were rinsed in 70% ethanol between extracting successive samples. The distribution of bug infection with *T. cruzi* at baseline has been previously reported in area 1 [36], across 0–51 MPI in area 2 [44] and across 0–78 MPI in area 3 [43, 50]; most of these samples (except those in [50]) were examined by OM.

For molecular diagnosis using kDNA-PCR, triatomines were dissected, and rectal samples in individual ampoules were mixed with 50 μl of sterile water, followed by boiling 10 min; the DNA was then extracted using DNAzol® (Invitrogen, Thermo Fisher Scientific, Waltham, MA, USA) as previously described [45]. We used a hot-start PCR targeting a 330-bp amplicon of the kinetoplast minicircle employing Taq Platinum (Invitrogen, Thermo Fisher Scientific) polymerase for increased sensitivity. PCR products were run in a 2% agarose gel and the products visualized under UV using Gel Red® (Biotium, Hayward, CA, USA). kDNA-PCR was employed for all triatomines collected at endpoint (for improved sensitivity) and to increase sample sizes in earlier periods (restricted to triatomines not examined by OM). For the latter goal, we tested up to 10 randomly selected third-instar nymphs or later stages (mainly collected by timed searches or householders) from each domestic site; bugs collected by other methods were sometimes used to reach the 10-sample target, except in area 3 where a 14-sample target was set for domestic sites [50]. Any specimen that tested positive by OM or kDNA-PCR (or by both methods) was considered to be infected with *T. cruzi* regardless of any disagreement between methods; hence, any specimen that tested negative by both methods or by the only one used was considered to be uninfected.

### Data analysis

This study was performed in accordance with the STROBE checklist (Additional file 2: Text S1). The selected transmission indices include: (i) the prevalence of triatomine infection with *T. cruzi*; (ii) the relative abundance of *T. cruzi*-infected *T. infestans* per unit effort; and (iii) the presence or prevalence of houses with ≥ 1 *T. cruzi*-infected *T. infestans*. For general descriptive purposes, all indices were computed at the household level (e.g. Fig. 1) and averaged by operational area, major vector habitats (described below) and time postintervention or intervention period. For more specific comparisons, the prevalence of infected bugs was computed for triatomines collected in domiciles, kitchens or storerooms (i.e. excluding chicken coops) by any method. Bugs collected in domiciles, kitchens or storerooms and examined for infection were pooled to increase sample sizes at the household level and because bug infection differed little between these ecotopes within each area and intervention period (see Results). Chicken coops and similar habitats used by non-competent hosts were excluded as they rarely harbored any infected bug in the study areas and elsewhere [37, 55, 56]. Abundance of infected bugs at the household level was computed as the product between domestic bug abundance (as determined by timed searches per unit effort) and the prevalence of infected bugs in domiciles, kitchens or storerooms, unless otherwise noted. Similarly, the proportion of houses with at least one infected bug was computed as the product between house-level domestic infestation (as determined by timed searches) and the presence of bug infection in said ecotopes (i.e. two binary variables). Composite metrics took the value of 0 in houses where either or both parameters were 0, and were assigned a missing value when information for one or both parameters was missing except when bug infection prevalence was 0, in which case the outcome was 0. The Agresti-Coull method was used to compute 95% confidence intervals (95% CI) for proportions.

We used the Mantel–Haenszel (MH) Chi-square (*χ*^2^) test to examine the association between the baseline presence of bug infection (yes/no) in domestic habitats, kitchens or storerooms and the future prevalence (or house-level presence) of bug infection in these habitats stratified by intervention period (early and late). Similarly, we used the MH test to compute an adjusted odds ratio (OR) of bug infection for a 1-unit increase in the frequency of occurrence of bug infection across the 9-year period (for both variables) stratified by area.

We fitted individual *T. cruzi* infection data in *T. infestans* collected in domiciles, kitchens and storerooms (response variable) clustered by house compound to a random-intercept multiple logistic regression model to address the fact that triatomines sharing the same exposures to infected hosts likely had dependent responses; housing units were assumed to be independent. The explanatory variables (categorical and binary) had been established a priori based on available empirical evidence: (i) operational area, three levels (areas 1–3), based on the large between-area variations recorded in the exploratory survey [41] and excluding area 4, which had sparser sample coverage; (ii) intervention period (3 levels: preintervention or baseline (0 MPI, coded 0), early (4–28 MPI, coded 1) and late period (34–100 MPI, coded 2); (iii) bug stage (5 levels: third-instar nymphs, fourth instars, fifth instars, adult males and adult females) [37, 57, 58]; (iv) household ethnicity (two levels: Creole and Qom) [38, 39]; (v) and baseline domestic infestation with *T. infestans* by timed manual searches, which correlates positively with triatomine and host infection and bug abundance in domestic habitats [22, 58]. These predictors posed no multicollinearity problems, as determined by variance inflation factors, *R*^2^ and condition numbers. Two-way interaction terms between intervention period and ethnicity or area were added one by one to the model and retained if the likelihood ratio test of the nested models had *P* < 0.05. We extended this model to verify that ecotope (i.e. domicile vs kitchens or storerooms) and its two-way interaction with intervention period and household ethnicity were insignificant at the 5% level; to simplify the exposition these results are not shown. Data management and statistical analyses were conducted using Stata version 15.1 [59]. Model fit was mainly assessed by the area under the receiver operator curve (AUC) using the package ‘ResourceSelection’ [60]; for the H-index, sensitivity and specificity, we used the package ‘hmeasure’ [61], both implemented in R.

We used random labeling of parasite-based indices to test the null hypothesis of random occurrence of events among the fixed spatial distribution of all houses. Global spatial analyses used the *L*(*r*) function implemented in ‘spatstat’ package [62]; *L*(*r*) values were computed from the mean of 25 replicates of 999 Monte Carlo simulations. The radial distances selected were 400, 1000, 2000 and 5000 m, approximately reflecting effects within the house-compound level, within or between villages (1000–2000 m) and at the area level, respectively. We considered three intervention periods to improve the simultaneous coverage of parasite-based indices across all four areas as explained above. The spatial aggregation of transmission indices was visualized via heat maps (i.e. density maps) using a kernel density estimation algorithm within the selected radial distances in QGIS 3.28.3. For illustration purposes, only the outcome for 2000 m is shown. The geographical coordinates of each housing unit were transformed to preserve anonymity. Individual triatomine-level data are given in Additional file 3: Table S1.

## Results

### Triatomine infection data

Of 6397 third-instar nymphs and later stages of *T. infestans* examined for infection across the 9-year period, 19.4% were infected with *T. cruzi* (Table 1). Overall, 5825 *T. infestans* were assessed by OM and 709 by kDNA-PCR. Among 129 T*. infestans* collected in area 1 at baseline and examined by both methods, 59 (45.7%) were co-positive, 46 (35.7%) were co-negative, 19 (14.7%) were OM-negative and kDNA-PCR-positive and five (3.9%) were OM-positive and kDNA-PCR-negative. Most of the examined triatomines were collected by timed manual (65.1%) or post-timed searches (23.4%) while a few were caught during or after insecticide spraying (7.2%) and by householders (4.4%).

**Table 1:** Distribution of *Triatoma infestans* collected by any method (including third instars and later stages) and infected with *Trypanosoma cruzi* according to operational area, main ecotopes, household ethnicity, triatomine stage, baseline domestic infestation (by timed manual searches) and intervention period in Pampa del Indio, 2007–2016

Factor	Level	No. of bugs collected	No. of bugs examined	Percentage of infected bugs^a^	
				Mean	95% CI
Area	1	5795	4050	19.5	18.3–20.8
	2	2016	611	14.9	12.3–17.9
	3	2945	1058	26.0	23.4–28.7
	4	2217	678	12.5	10.2–15.3
Ecotope	Domiciles	6252	3164	27.2	25.7–28.8
	Kitchen-storerooms	3845	2090	16.6	15.1–18.3
	Chicken coops	2682	1094	1.8	1.2–2.8
	Missing data	194	49	24.5	
Household ethnicity	Qom	5689	2206	26.9	25.1–28.8
	Creoles	7234	4168	15.3	14.2–16.4
	Missing data	50	23	39.1	
Stage	3	2645	640	5.9	4.3–8.1
	4	2058	1050	9.9	8.2–11.9
	5	3416	2038	20.6	18.9–22.4
	Adult females	2184	1156	27.6	25.1–30.2
	Adult males	2664	1513	23.8	21.7–26.0
	Missing data	6	0		
Baseline domestic infestation	Yes	7308	3905	26.8	25.5–28.2
	No	5063	2376	7.2	6.2–8.3
	Not applicable	602	116	19.8	
Intervention period	Baseline	8131	4115	24.1	22.8–25.4
	Early	2720	1584	10.1	8.7–11.7
	Late	2122	698	13.0	10.6–15.8
Total		12973	6397	19.4	18.4–20.4

*CI* Confidence interval,* Creole* person of European [mainly Spanish] or mixed origin),* Qom *indigenous people

^a^Infection with *Trypanosoma cruzi* determined by optical microscopy (OM) or kinetoplast DNA (kDNA)-PCR

The unadjusted prevalence of *T. cruzi* infection in *T. infestans* varied widely among areas, ranging from 12.5% (area 4) to 26.0% (area 3), peaked in domiciles (27.2%) and kitchens or storerooms (16.6%) and was rare in chicken coops (1.8%) (Table 1). Chicken coops produced 20 infected triatomines (including 4 fifth instars, 6 adult females and 10 adult males) in 11 houses (all but one were Creole households in area 1 at 0 MPI); in six of these 11 houses, bug infection and infestation co-occurred elsewhere within the same compound. Bug infection was nearly twofold higher in Qom (26.9%) households than in Creole households (15.3%), and increased with increasing bug stage from 5.9% among third instars to 23.8–27.6% among adult triatomines, and when a domestic infestation was detected by timed searches at baseline (26.8%) rather than not (7.2%). Bug infection fell from a maximum (24.1%) at baseline to 10.1% and 13.0% over the early and late intervention periods, respectively (Table 1). The endpoint survey revealed the all-time minimum prevalence of bug infection (0.9%), as determined by kDNA-PCR only. Of the 1241 infected bugs detected throughout the study, 990 (79.8%) occurred at baseline. In 15 houses from area 1 with bugs tested both in the exploratory (September) and baseline (October-December) surveys before insecticide spraying, the prevalence of infection varied little, from 59.5% (91/153; 95% CI 51.6–66.9%) to 49.3% (134/272; 95% CI 43.4–55.2%), respectively.

The sampling fraction of triatomines examined for infection averaged 59.1% (range: 40.5–74.4%) across areas among those collected by timed or post-timed searches in domiciles, kitchens or storerooms. The mean number of triatomines collected per house by any method and tested was 14.7 (95% CI: 12.0–17.4). The size of this group sample would be approximately sufficient to detect at least one infected bug with probability 0.90 when the minimum prevalence of infection was 20–30% if the pathogen were present, domestic bug population size (of third instars and later stages) exceeded 30, test sensitivity and specificity was 0.95 and the probability of incorrectly classifying a pathogen-free population as infected was no greater than 0.05 [63: p. 675].

Random-intercept multiple logistic regression analysis of bug infection revealed statistically significant effects of intervention period, operational area, household ethnicity, bug stage and baseline domestic infestation and significant two-way interaction terms between intervention period and area or household ethnicity (Wald *χ*^2^ = 161.1; *df* = 16; *P* < 0.001; 4594 observations clustered in 309 houses, each averaging 14.9 observations [range 1–351]) (Table 2). The model had an excellent classification performance (AUC = 0.916), with 83.0% accuracy, 84.7% sensitivity and 82.5% specificity, and H-index = 0.519. The intraclass correlation coefficient (rho) was 0.52 (95% CI 0.44–0.61), indicating significant residual outcome variation at the household level. The adjusted odds of bug infection strongly decreased as the intervention period progressed depending on operational area and household ethnicity. The predicted marginal effects of intervention period and ethnicity on the mean probability of bug infection are shown in Additional file 4: Figure S2A. The latter in a Qom household remained at 0.30–0.32 over the baseline and early intervention period and then dropped to 0.09 over the late period; in comparison, in a Creole household the predicted probability of bug infection plummeted from 0.22 after the attack phase and remained at 0.06–0.09 thereafter. The predicted marginal effects of intervention period and operational area are shown in Additional file 4: Figure S2B. The mean probability of bug infection remained high over the baseline (0.28) and early (0.21) period before dropping to 0.10 in area 1, whereas the predicted bug infection fell to < 0.09 after the baseline period in areas 2 and 3. Baseline domestic infestation and bug stage also exerted strong positive effects. Restricting the analysis to houses with ≥ 10 triatomines examined for infection reduced the log-likelihood substantially but did not modify the qualitative results obtained above (Wald *χ*^2^ = 144.7; *df* = 16; *P* < 0.001; 4081 observations clustered in 123 houses).

**Table 2:** Results of random-intercept multiple logistic regression analysis of *Trypanosoma cruzi* infection in *Triatoma infestans* collected in domiciles, kitchens or storerooms across operational areas in Pampa del Indio, 2007–2016

Regression term	Levels	OR	95% CI, lower limit	95% CI, upper limit	*P*
Area	2	0.92	0.24	3.53	0.90
	3	0.51	0.23	1.14	0.10
Intervention period	Early	0.18	0.07	0.46	< 0.001
	Late	0.24	0.08	0.70	0.009
Area × Intervention	2 × Early	0.11	0.02	0.80	0.03
	2 × Late	0.12	0.02	1.00	0.05
	3 × Early	0.40	0.07	2.32	0.31
	3 × Late	1.52	0.43	5.36	0.52
Household ethnicity	Qom	2.47	1.14	5.40	0.02
Intervention × Ethnicity	Early × Qom	8.21	2.45	27.53	0.001
	Late × Qom	0.35	0.10	1.27	0.11
Stage	4	1.77	1.05	3.01	0.03
	5	5.59	3.02	10.34	< 0.001
	Adult females	13.22	6.86	25.47	< 0.001
	Adult males	10.99	5.57	21.68	< 0.001
Baseline domestic infestation	Yes	3.37	1.79	6.35	< 0.001
Intercept		0.010	0.004	0.023	< 0.001

Reference levels: area, area 1; intervention period, baseline (0 months postintervention [MPI]); household ethnicity, Creole; bug stage, third instar; domestic infestation, not infested as determined by timed searches

* Qom *Indigenous people, *OR* odds ratio

### Area-wide transmission indices

The time series of transmission indices by area are shown in Fig. 1. The prevalence of bug infection tended to decline and varied widely over time (Fig. 1a). Conversely, both the proportion of houses with at least one infected bug and mean infected-bug abundance plummeted immediately after the attack phase and tended to slowly decline postintervention (Fig. 1b, c), when the former never exceeded 1.3% and the latter was < 0.3 infected bugs per unit effort barring one occasion. The overall prevalence of infestation at baseline was moderate both at the house (26.8%) and domestic (19.0%) levels among 1172 houses inspected by timed searches [41]. House infestation dropped immediately after the attack phase in areas 2 and 3, where it never recovered. It reached the initial control target (5%) at 22 MPI in area 1, where maximum levels of house infestation (41.4%) and triatomine abundance occurred preintervention, and no further surge was revealed thereafter. Conversely, in area 4, both house infestation and bug abundance fell after the attack phase, recovered by 40 MPI and plummeted by endpoint (Fig. 1d, e).

All five transmission indices were significantly and positively correlated across areas. The weakest correlation coefficients were between mean bug infection prevalence and mean bug abundance (*r* = 0.45, *df* = 29, *P* = 0.01) or mean house infestation (*r* = 0.51, *df* = 29, *P* = 0.004). Conversely, the correlations between bug infection prevalence and the proportion of houses with at least one infected bug (*r* = 0.63, *df* = 29, *P* < 0.001) and infected-bug abundance (*r* = 0.62, *df* = 29, *P* < 0.001) were stronger. All other correlation coefficients involving parasite-based indices were more strongly related to mean house infestation and bug abundance (*r* = 0.88–0.98, *df* = 29, *P* < 0.001).

### Spatial analysis

**Fig. 2:**
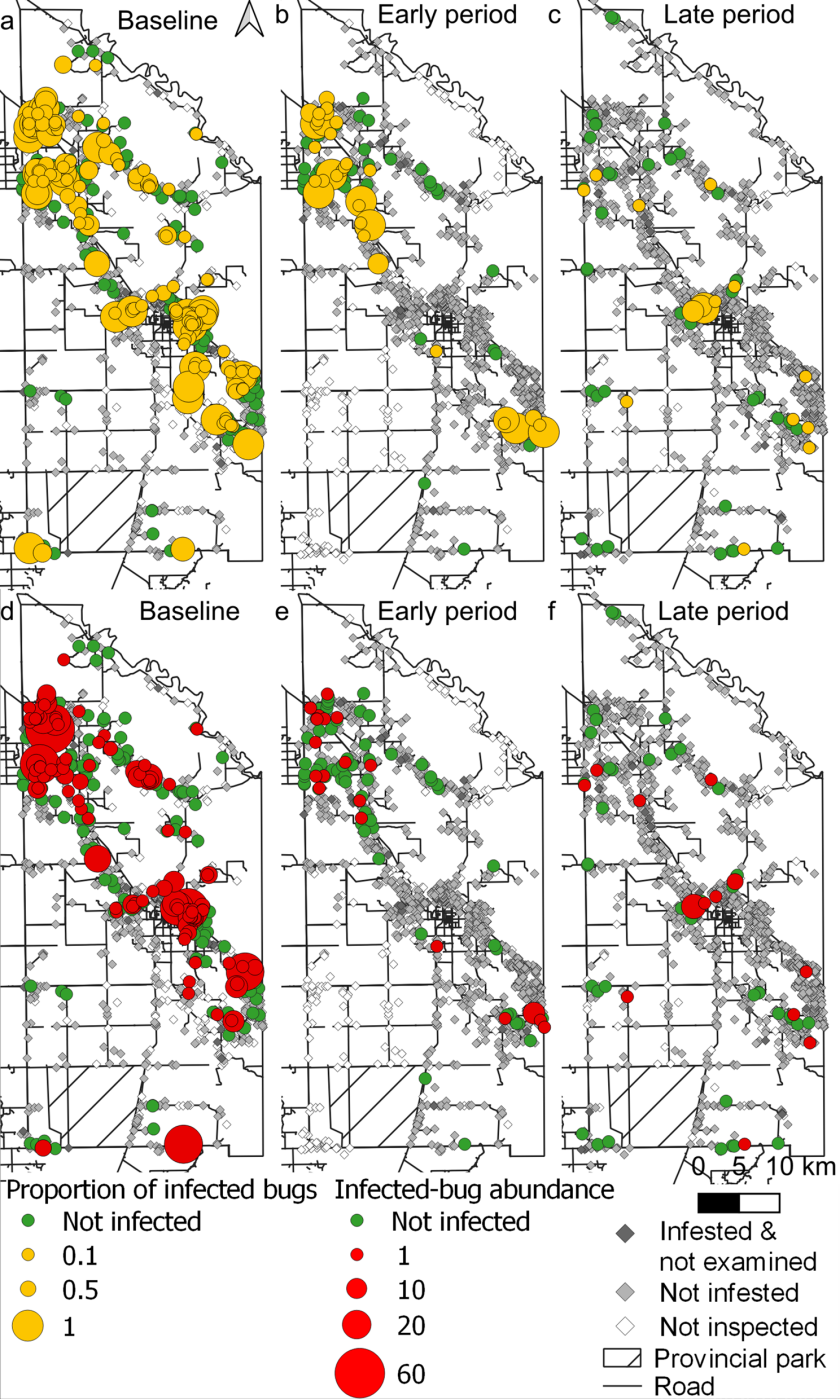
Distribution of the prevalence (**a**–**c**) and abundance (**d**–**f**) of *Triatoma infestans* infected with *Trypanosoma cruzi* according to intervention period (early or late) in Pampa del Indio 2007–2016. **a** Baseline (0 MPI), **b** early intervention period (4–28 MPI), **c** late intervention period (34–100 MPI). MPI, Months postintervention

The spatial distributions of the house-level prevalence of bug infection and infected-bug abundance are shown in Fig. 2. At baseline, bug infection was widely dispersed across the district, except in sparsely populated sections in the southwest part, with some indication of heterogeneity (Fig. 2a). The interventions over the next 2 years largely reduced triatomine abundance and suppressed bug infections except in two large clusters in the northwestern and southeastern parts (Fig. 2b). The late intervention period exhibited scattered bug infection foci adjacent to prior aggregates of bug infection and (re)emergent foci in the core section, in the proximity to peri-urban and urban areas (Fig. 2d). The abundance of infected bugs (Fig. 2d–f) and the presence of ≥ 1 *T. cruzi*-infected bugs in a house (Additional file 4: Figure S2) displayed similar waning patterns over time postintervention as bug infection prevalence (Additional file 5: Figure S3).

Global spatial analysis revealed: (i) a significant aggregation of the presence of triatomine infection at the household level at baseline within all tested radii except 5000 m; (ii) a significant aggregation within distances from 2000 to 5000 m over the early intervention period; and (iii) no aggregation after the early intervention period (Additional file 6: Figure S4). For the global spatial analysis at 400–1000 m radii, aggregation levels decreased over time postintervention. Aggregation of abundance of infected bugs was observed only at baseline from 2000 to 5000 m (Additional file 7: Figure S5).

**Fig. 3:**
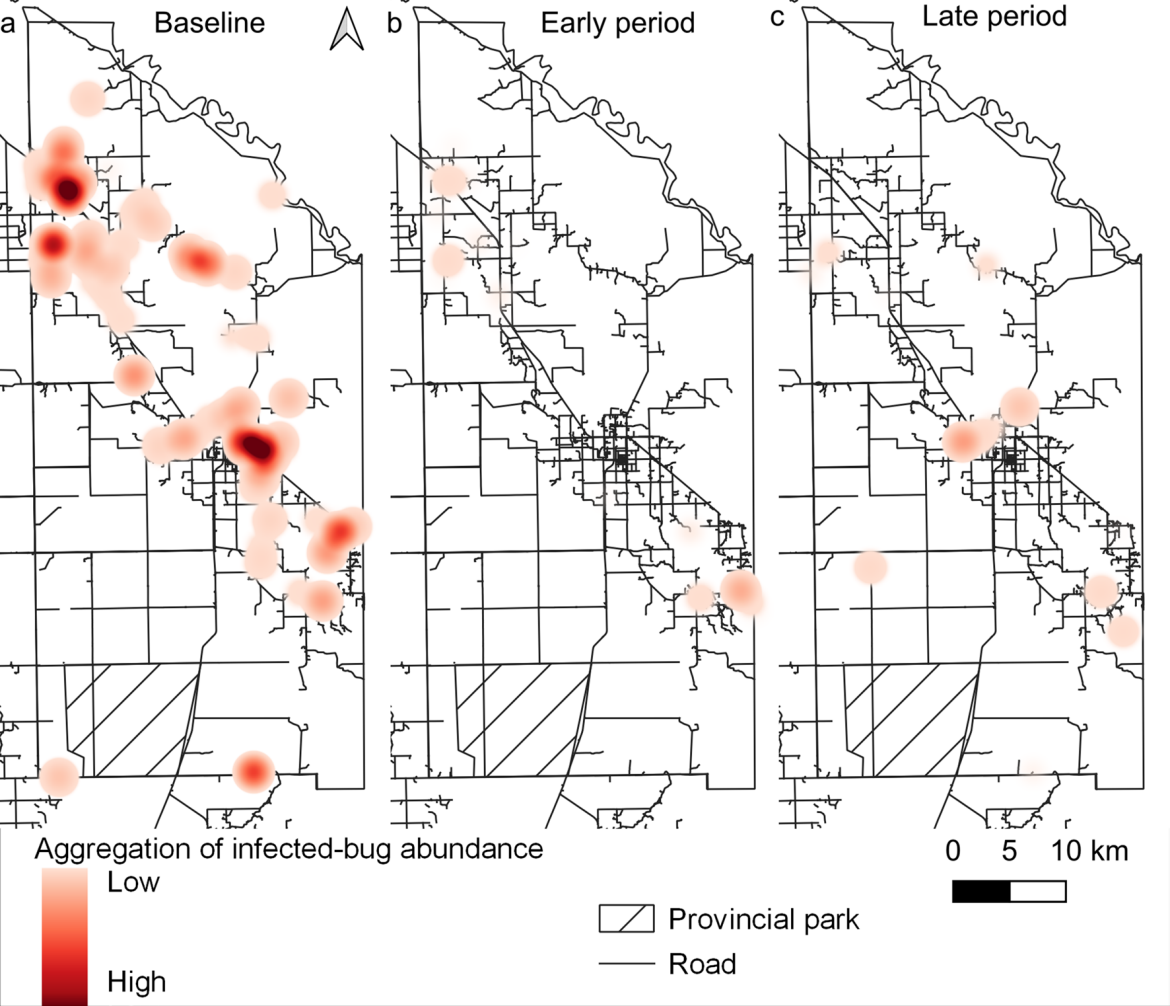
Heatmaps of the relative abundance of *Triatoma infestans* infected with *Trypanosoma cruzi* within 2000 m according to intervention period (early or late) with area-wide insecticide spraying in Pampa del Indio, 2007–2016. **a** Baseline (0 MPI), **b** early intervention period (4–28 MPI), **c** late intervention period (34–100 MPI). MPI, Months postintervention

The time series of heatmaps showed a sharp change in the location, frequency and intensity of the aggregation of abundances of infected bugs after the attack phase; Fig. 3 illustrates the patterns observed at distances of 2000 m over the three intervention periods. Preintervention clusters occurred in the northwestern part across area 1, in the district core (around the main town and its sprawls) and in the southeastern part including sections of areas 2 and 3 (Fig. 3a). These clusters disappeared or waned after spraying with pyrethroid and malathion (Fig. 3b) and persisted with less intensity or reappeared in the core during the late intervention period, as in area 4, which had sparser surveillance-and-response operations (Fig. 3c).

### Occurrence of triatomine infection

**Fig. 4:**
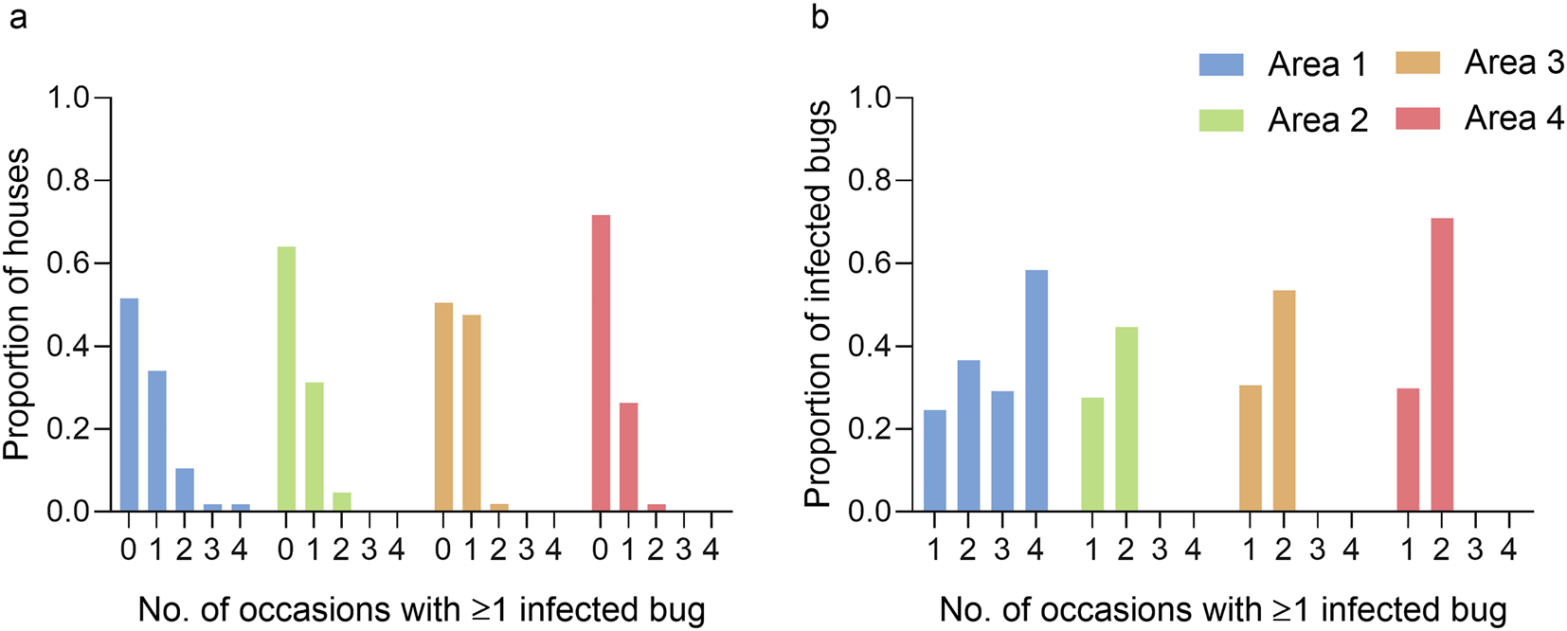
Frequency distribution of survey occasions (occurrences) (**a**) in which at least 1 *Trypanosoma cruzi*-infected* Triatoma infestans* was collected by any method in domiciles, kitchens or storerooms, and (**b**) prevalence of* T. cruzi* infection in* T. infestans* collected in the same ecotopes, by area in Pampa del Indio over 2007–2016

The longitudinal design of the study allowed us to compute the frequency of survey occasions (i.e. occurrence) in which at least one *T. cruzi*-infected *T. infestans* was detected in domiciles, kitchens or storerooms across the 9-year period (Fig. 4a). No infected bug was ever detected in 56.2% of 379 houses with triatomines examined for infection. Bug infection was observed once (34.2%), twice (10.6%) and on 3–4 occasions (3.7%) across the 13 surveys conducted in area 1. In areas 2–4, which were surveyed for triatomines 5–8 times, bug infection was detected once (range 26.4–47.5%) and in no more than two occasions in 1.9–4.7% of houses.

The prevalence of *T. cruzi* infection in *T. infestans* increased with increasing frequency of occurrence of bug infection at the household level across the 9-year period (Fig. 4b). In area 1, bug infection prevalence steadily increased from 24.6% to 58.4% when the occurrence of bug infection rose from one to four occasions. In other areas, when bug infection increased from once to twice, the prevalence of bug infection increased from 27.6% to 44.7% in area 2; from 30.6% to 53.5% in area 3 and from 29.9% to 71.0% in area 4 (Fig. 4b). The relative odds of bug infection stratified by area was 1.70-fold (95% CI: 1.55–1.86) higher for a 1-unit increase in the occurrence of bug infection from 1 to 4 (MH *χ*^2^ = 124.8, *df* = 1, *P* < 0.001), with significant heterogeneity among areas (homogeneity *χ*^2^ = 16.5, *df* = 1, *P* = 0.001); area-specific ORs rose from 1.61 (area 1), 2.15 (area 2), 2.79 (area 3), to 6.00 (area 4).

**Fig. 5:**
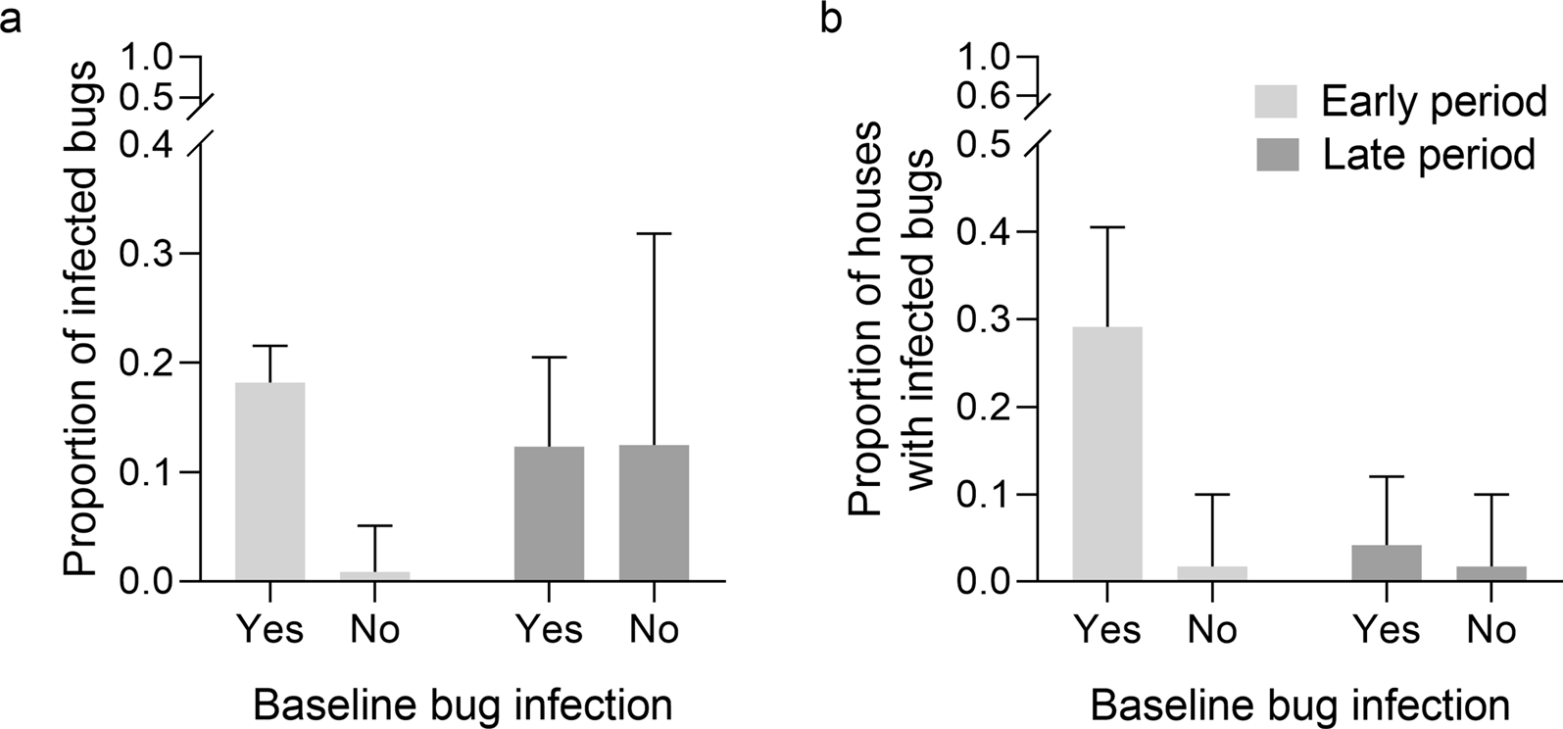
Association between the baseline presence of *Trypanosoma cruzi*-infected *Triatoma infestans* in domestic habitats, kitchens or storerooms and the future prevalence of infected bugs (**a**) or the future proportion of houses with ≥ 1 infected bugs (**b**) over the early (4–28 MPI) and late (34–100 MPI) intervention periods in Pampa del Indio area 1, 2007–2016. MPI, Months postintervention

We then asked whether the baseline presence of bug infection (yes/no) in domestic habitats, kitchens or storerooms was positively associated with the future prevalence of *T. cruzi* infection in *T. infestans* collected by any method in said habitats over the early (4–28 MPI) and late (34–100 MPI) intervention periods in area 1. During the early period, 18.2% (108/592) of the triatomines collected in baseline-positive houses were infected, whereas only 0.8% (1/118) of those from baseline-negative houses were infected (Fig. 5a). Bug infection over the late intervention period was unrelated to whether the house had ≥ 1 infected bugs at baseline (12.4%, 12/97) or not (12.5%, 3/24). The relative odds of bug infection postintervention stratified by intervention period was 7.13-fold (95% CI: 2.58–19.69) greater when the house had ≥ 1 infected bugs at baseline rather than when it had not (MH *χ*^2^ = 19.6, *df* = 1, *P* < 0.001). The homogeneity of ORs between strata was rejected (homogeneity *χ*^2^ = 13.0, *df* = 1, *P* < 0.001), indicating differential effects of baseline infection status between subsequent intervention periods.

A stronger association was obtained between baseline bug infection and the future presence/absence of bug infection with *T. cruzi* at the household level (rather than bug infection prevalence) in area 1 (Fig. 5b). Over the early period, a large fraction of the baseline-positive houses (29.2%, 21/72) subsequently harbored ≥ 1 infected triatomines, whereas only 1.7% (1/58) of baseline-negative houses had ≥ 1 infected bugs. Bug infection over the late period dropped in baseline-positive houses (4.2%, 3/72) relative to previous levels and remained low in baseline-negative houses (1.7%, 1/58). The relative odds of a house with ≥ 1 infected bugs postintervention was 11.40-fold (95% CI: 2.60–49.93) greater when the house had ≥ 1 infected bugs at baseline rather than when it had not, excluding houses with no bug examined for infection (MH χ^2^ = 16.7, *df* = 1, *P* < 0.001; homogeneity* χ*^2^ = 2.47, *df* = 1, *P* = 0.12).

**Fig. 6:**
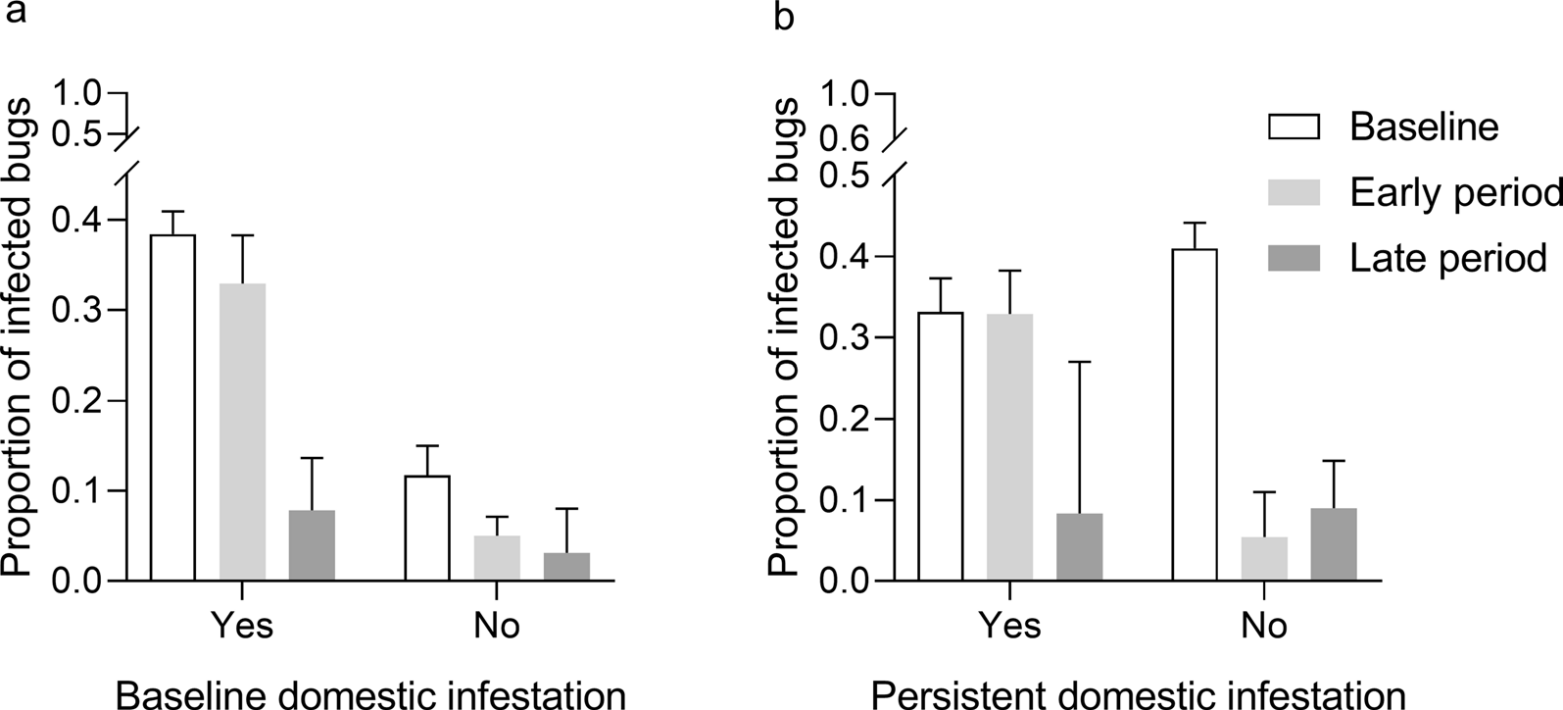
Prevalence of *Trypanosoma cruzi* infection in *Triatoma infestans* collected in domiciles, kitchens or storerooms at baseline (0 MPI) and in the early (4–28 MPI) and late (34–100 MPI) intervention period according to baseline domestic infestation (**a**) and persistent domestic infestation (**b**) over 0–28 MPI in Pampa del Indio area 1, 2007–2016. Domestic infestation at household level was determined by timed searches. MPI, Months postintervention

We tested whether the prevalence of *T. cruzi* infection in *T. infestans* collected in domiciles, kitchens or storerooms across intervention periods was associated with a baseline and persistent domestic infestation detected by timed searches in area 1 (Fig. 6). Over each period, bug infection was consistently greater if a domestic infestation had been detected at baseline (Fig. 6a). The relative odds of bug infection stratified by intervention period was 5.5-fold (95% CI: 4.28–7.06) greater when a domestic infestation was detected rather than when it was not (MH *χ*^2^ = 224.8, *df* = 1, *P* < 0.001), with weakly significant heterogeneity between periods (homogeneity *χ*^2^ = 7.3, *df* = 1, *P* = 0.03). In houses with a persistent domestic infestation (Fig. 6b), the fraction of infected bugs remained virtually invariant between the baseline (33.1%, 170/513) and early period (32.9%, 104/316), and then fell to 8.3% (2/24). Conversely, in houses with a non-persistent domestic infestation, bug infection plummeted between the baseline (41.0%, 390/951) and the early (5.4%, 7/129) or late periods (9.0%, 13/145). The relative odds of bug infection stratified by intervention period was 6.0-fold (95% CI 2.99–11.95) greater when a persistent domestic infestation was detected rather than when it was not (MH *χ*^2^ = 33.3, *df* = 1, *P* < 0.001), with significant heterogeneity between periods (homogeneity *χ*^2^ = 8.0, *df* = 1, *P* = 0.005), indicating that most effects occurred between the baseline and the early intervention periods.

**Fig. 7:**
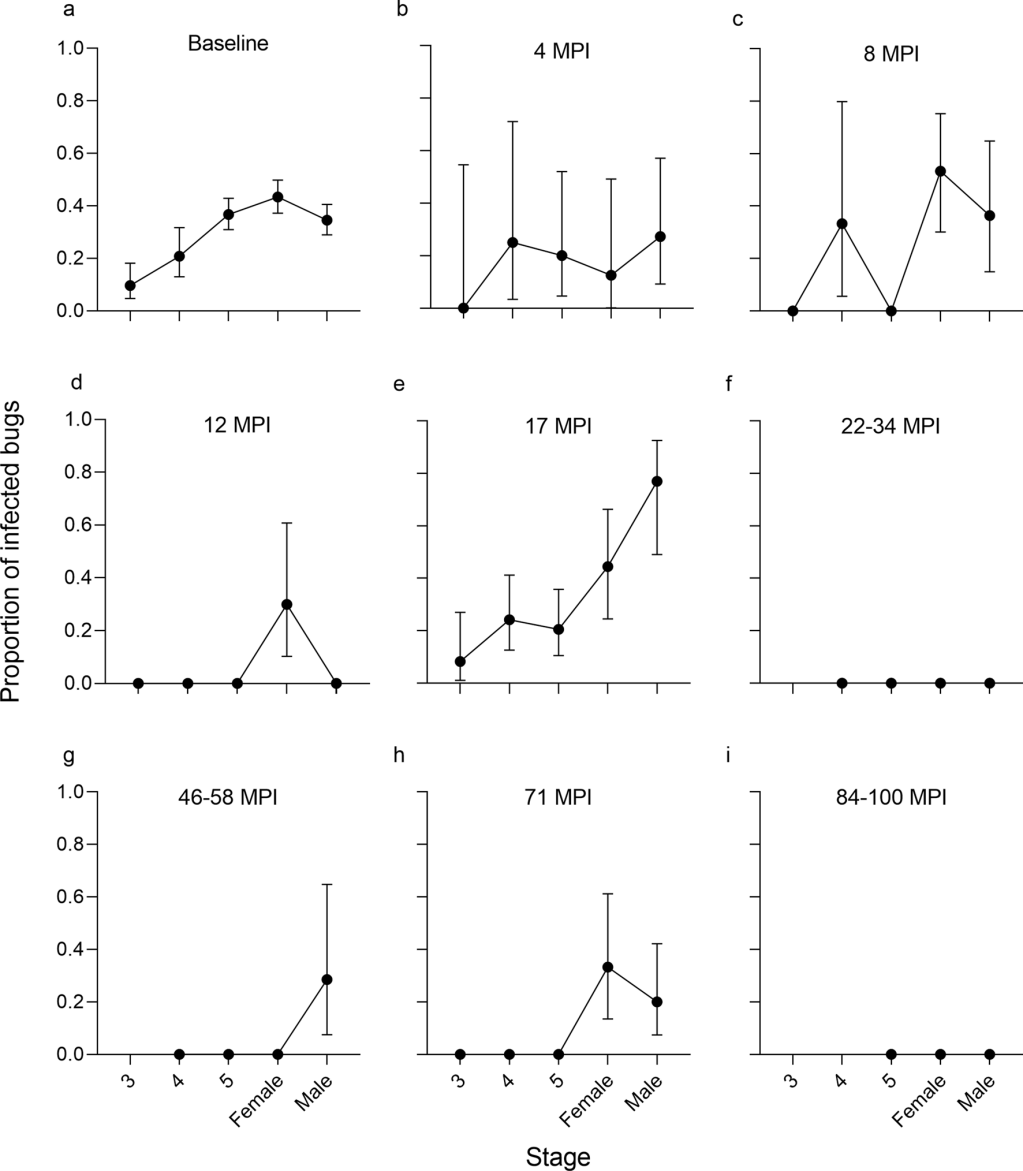
**a**–**i** Stage-specific prevalence of *Trypanosoma cruzi* infection in *Triatoma infestans* collected in domiciles according to time (in months) postintervention in Pampa del Indio area 1, 2007–2016. Vertical bars: 95% Confidence intervals. 3, 4, 5, Third, fourth, fifth instar nymphs, respectively; MPI, months postintervention

The temporal trends in stage-specific prevalence of *T. cruzi*-infected *T. infestans* in area 1 domiciles are shown in Fig. 7. Before interventions, domestic bug infection increased steadily with increasing stage from 13.3% in third instars to 39.3% and 46.7% in adult males and adult females, respectively (Fig. 7a). The earliest stage found to be infected at baseline was the third instar. The infection curve flattened over 4–8 MPI (when fourth instars were the earliest stage infected), and the trend disappeared at 12 MPI, when only adult females were infected (Fig. 7d). The curve recovered a positive trend at 17 MPI (late summer-early fall), when third instars were again the earliest stage infected. Infection was nil over 22–34 MPI (Fig. 7f) and became rare thereafter and restricted to a few adult triatomines (Fig. 7g-i).

## Discussion

The mixed-effects multiple regression model revealed the important effects of the intervention period, baseline domestic infestation, household ethnicity, bug stage and area on the odds of bug infection with *T. cruzi*, showing excellent classification performance. Bug infection strongly dropped after the attack phase (while the earliest infected stage increased) as surveillance-and-response operations progressed from the early to the late intervention period in which *T. infestans* was nearly suppressed across the district [41]. While the initial attack phase killed most of the extant triatomines (including the infected ones), the selective treatments most likely suppressed a fraction of those occurring in persistent, pyrethroid-resistant foci (in which the larger stages may survive better) or all triatomines in pyrethroid-susceptible foci, and did not allow their offspring to survive for a long enough period to become infected. In-migration of infected *T. infestans* from other houses within the district was likely minimized as the attack phase achieved nearly full coverage in a short time period within each area. The borders with neighboring districts (all infested) and the time lag between attack phases in adjacent areas (10–25 months) allowed some room for triatomine spill-over, but in practice, the relevant sections displayed no apparent excess of infected bugs postintervention (Figs. 2, 3).

Parasite-based and infestation indices were significantly and positively correlated and remained depressed shortly after the attack phase. However, the prevalence of bug infection displayed an overdispersed pattern influenced by the few low-density infestations detected and the few triatomines examined for infection, thus giving a misleading impression of high-risk status at the area-wide level. Conversely, the abundance of infected bugs and the proportion of houses with ≥ 1 infected bugs provided information from all houses inspected by timed searches (including those negative for *T. infestans*) and were better summary measures of area- or village-wide risk status than bug infection prevalence (Fig. 1a–c). Both composite transmission indices revealed the mid-term surge in area 4, whereas house-level infestation and bug abundance were more influenced by focal or light infestations anywhere within the house compound and conveyed no explicit information on triatomine infection with *T. cruzi*.

Three large sources of uncertainty argue against assigning an absolute value to point estimates of any of these indices: (i) the limited sensitivity of timed-manual searches and OM-based diagnosis (see below); (ii) the vagaries of *T. cruzi* transmission by contamination with triatomine feces, including its apparent inefficiency [64]; and (iii) lack of routine data on triatomine blood-feeding rates and contact with humans, with these latter two parameters displaying large spatial and temporal variations in the few studies that have measured both [27, 65, 66]. Notwithstanding these caveats, the biased estimates returned by timed searches or OM would still provide internally valid measures of relative change as long as bias remained approximately constant over time [31]; whether the detection bias holds approximately constant postintervention is uncertain and more research on this respect is needed. Infestation- and parasite-based indices are best used for comparative purposes and trend detection in population-based monitoring programs rather than as point or absolute estimates.

The occurrence and spatial distribution of bug infection suffered major changes after the attack phase, which virtually destroyed the existing pattern of aggregation of abundances of infected bugs and other transmission indices at baseline (Fig. 3a, b). The intervention period represents cumulative surveillance-and-response efforts and other context effects, including a declining pressure of house infestation from residual bug foci, a declining prevalence of *T. cruzi*-infected dogs and cats due to their fast population turnover [37, 49], an increasing fraction of non-infected children as a consequence of large annual birth rates (3.1% in area 3 over 2012–2015) in a fast-growing population not exposed to vector-borne transmission and mass etiologic treatment of *T. cruzi*-seropositive children [53] (subsequently scaled up to areas 2–4). Thus, the seroprevalence of *T. cruzi* infection in children aged  < 16 years dropped from a frequency of 10.6–27.7% at baseline to 2.5% at endpoint, with no incident human case detected, while dog infection fell from 26.0% (in area 1, where transmission was most intense) to 3.1% across areas at the endpoint [49]. In other domestic locations infested with *P. megistus* and *T. infestans*, the prevalence of *T. cruzi*-infected triatomines substantially increased with the household presence and numbers of *T. cruzi*-seropositive children and seropositive dogs or cats before control actions [58, 67]. Moreover, human infectiousness to triatomine bugs significantly declined with increasing age of host [68–71], whereas dog infectiousness was age-independent and highly aggregated [72]. Consequently, *T. cruzi*-seropositive children and dogs or cats are important domestic reservoir hosts in areas with recent or current vector-borne transmission while untreated seropositive adult people remain as long-term parasite sources, sometimes as infectious as seropositive children [71]. Whether triatomine human-feeding contacts are distributed at random among all resident human hosts or whether children are preferentially selected is unknown; knowledge on this aspect of transmission is therefore important for a mechanistic understanding of changing bug infection rates under sustained control efforts.

The observed negative time trend in bug infection in Pampa del Indio is in agreement with the outcomes of other control efforts directed to *T. infestans* and appears to be generalizable to the study region and beyond. In the dry Argentine Chaco, following sustained surveillance-and-response efforts with pyrethroids, triatomine infection plummeted from 49.1% to a mean of 5.6% over the subsequent 12 years [26: Supplementary Information Figure 5], a trend paralleled by child and dog infection. *Trypanosoma cruzi* infection in domestic *T. infestans* also declined after pyrethroid spraying and selective treatments over a 3-year period [73], across control efforts that were sustained rather than pulsed over several years [55] and across a rural-to-urban gradient [74]. In Paraguari (Paraguay), house spraying with a pyrethroid alone or combined with housing improvement suppressed *T. cruzi* infection in *T. infestans* over an 18-month period [75]. Similar long-term declines in bug infection after sustained insecticide treatments were recorded in Goiás, Brazil [76, 77]. In a field trial of fluralaner administered to dogs, both the prevalence of *T. infestans* infected with *T. cruzi* and the abundance of infected bugs fell postintervention and remained marginal or nil over a 2-year period [78].

The strong association between baseline domestic infestation or baseline bug infection and subsequent bug infection indicates that a rather small, identifiable subgroup of households were under an increased transmission risk before and after interventions. Similarly, the prevalence of bug infection increased with a persistent domestic infestation at the household level and the frequency of occasions in which bug infection was detected. These results are consistent with several pieces of evidence collected elsewhere: (i) domestic infestation and domestic bug infection were positively associated before and after sustained control efforts [22: Figure 1; 58: Figure 2]; (ii) domestic infestation and abundance of infected bugs at baseline were strongly and positively related to the household frequency of humans and dogs infected with *T. cruzi* [33, 34, 37–39]; and (iii) preintervention and postintervention house infestation with *T. infestans* were positively associated across several locations, including Pampa del Indio [9, 41]. In the current study, 19% of all housing units inspected by timed searches at baseline had a domestic triatomine infestation, and 56.2% had at least once ≥ 1 infected bugs across the 9-year period. Temporal aggregation of bug infection also occurred, as 80% of all *T. cruzi*-infected triatomines detected over the 9-year period occurred preintervention. Hence, the fraction of houses at risk of vector-borne transmission was close to the 80–20 rule-of-thumb [79]. These results, probably driven by stable determinants of house infestation and parasite transmission linked to the household level, open new opportunities for sustainable vector control and disease prevention by focusing on the fraction at risk.

To our knowledge, this study has produced the first empirical data linking vector control failures to pyrethroid resistance, persistent infestation and persistent bug infection with *T. cruzi* in Triatominae. Several persistent foci that required selective re-applications of pyrethroids over 4–17 MPI were ultimately suppressed with malathion over 22–28 MPI [42, 54]. In the present study, we show that in houses with a persistent domestic infestation, the fraction of infected bugs remained virtually invariant over approximately 0–24 MPI (Fig. 5). Hence, persistent bug infection is another expression (and marker) of vector control failures associated with pyrethroid resistance, analog to those related to substrate- or dose-dependent poor residuality of insecticides (e.g. [80, 81]). Although high resistance confers a relative advantage in habitats under pyrethroid pressure, parasite transmission may perhaps become less efficient: pyrethroid resistance was found to affect several vital rates and to reduce the population growth [82, 83] and postfeeding defecation of *T. infestans*, jointly with the fraction of trypomastigotes produced relative to susceptible triatomines [84]. Key pieces of evidence for which there is contradictory (or no) information include whether pyrethroid resistance modifies triatomine blood-feeding rates and blood-meal size [84, 85].

In the present study, household ethnicity was a major driver of the odds of bug infection in interaction with the intervention period. These results are consistent with earlier reports of Qom households having a substantially greater domestic infestation and triatomine abundance than Creole households across areas and periods [41] and a greater prevalence of human, dog or cat and bug infection at baseline [37–39]. Conversely, Creole households have usually been reported to have consistently greater peridomestic infestation, more peridomestic structures and more domestic animals than Qom households [51, 52]. It remains unclear why the prevalence of bug infection after the attack phase took longer to fall in Qom compared to Creole households (Additional file 4: Figure S2A) and whether this pattern is somehow affected by the intense household mobility of Qom people in some district sections [43].

The prevalence of *T. cruzi* infection in *T. infestans* steadily increased with developmental stage, with fifth instars and adults showing maximal infection rates, as has been shown in other endemic areas before control efforts (e.g. [22, 37, 57, 58]). This result was expected based on: (i) the virtually irreversible nature of bug infection; (ii) larger average blood-meal size [70: Figure 2] and cumulative frequency of previous blood meals with increasing bug stage; and (iii) increasing chances of ever being exposed to an infectious hosts over the insect’s life span. In theory, bug infection curves may be affected by variations in triatomine susceptibility among developmental stages, pathogenicity of *T. cruzi* infection and recruitment of susceptible insects. *Trypanosoma cruzi* appears to be weakly pathogenic to triatomines depending on the presence of stressors (starvation, extreme temperature), parasite strain and intensity of bug infection [87]; hence, the parasite may affect triatomine vital rates to some extent. These parasite-related effects are beyond the scope of this study and the magnitude of its effects is uncertain.

All of the *T. cruzi*-infected *T. infestans*, with minor variations among areas and intervention periods, were virtually concentrated in domiciles and nearby peridomestic outhouses used as resting sites by dogs and cats. As nearly all of the human feeding contacts of *T. infestans* occur in domiciles [27, 56, 65, 87], the proximity of domiciles to nearby peridomestic structures combined with the dispersal ability of *T. infestans* [88–90] suggest these factors may be relevant for *T. cruzi* transmission. Conversely, chicken coops were large sources of uninfected chicken-fed triatomines [27, 37, 52, 55, 56]. The few infected *T. infestans* collected in chicken coops at baseline or shortly thereafter suggests insect dispersal within or between houses, as did the unusual finding of human-fed triatomines in chicken coops [66].

This study has a number of limitations. An important limitation from an inferential point of view is the lack of a control area, for obvious ethical reasons. Yet there is compelling evidence that established domestic infestations with *T. infestans* did persist in the absence of effective control actions (e.g. [41, 78, 91]), as did the occurrence and prevalence of *T. cruzi* infection in field populations of *T. infestans* [57, 58] and *P. megistus* [80, 81]. In our study, in the absence of insecticide spraying, bug infection matched by house remained at similarly high levels between the exploratory and baseline surveys in area 1. To overcome the absence of a control group we analyzed the data using a before-after design.

The limited sensitivity of timed manual searches and OM-based diagnosis affects the accuracy of infestation and parasite-based indices. Under our procedures, OM-based diagnosis of field-collected *T. infestans* failed to detect 19% of infections revealed by kDNA-PCR in Amamá [92] and 13% of kDNA-PCR-positive nymphs used in xenodiagnosis of *T. cruzi*-seropositive humans and dogs [71, 72]. In Pampa del Indio area 1 at baseline, OM missed 23% of *T. infestans* testing positive by either method and kDNA-PCR missed 6% of them, probably related to PCR inhibitors [92]. Our case definition of a *T. cruzi*-positive bug implicitly assumes that both methods have equivalent sensitivity and, consequently, it underestimates the true fraction of infected bugs. Since PCR-based diagnosis comprised 10.9% (395/3626) of all triatomines examined for infection at baseline and 100% (115/115) of those tested at endpoint, adjusting for the lower sensitivity of OM would produce a steeper negative time trend of bug infection across intervention periods.

In general, several PCR-based methods have largely performed better than OM-based diagnosis of *T. cruzi* in *T. infestans* [93, 94], although not always [95]. OM-based diagnosis is probably worse in sylvatic triatomines [96, 97], in which the intensity of infections tends to be lower than that in field-collected *T. infestans*, which were found to harbor tens of thousands of *T. cruzi* per microliter of rectal contents in late spring [57] and substantially fewer parasites at other times. A key unanswered question is whether triatomines with non-patent *T. cruzi* infections as determined by OM are of equal worth in terms of transmissibility as OM-positive insects. The large disagreement among results obtained with various PCR-based methods applied to triatomine infections with *T. cruzi* in the northern hemisphere supports the lack of a true gold standard [98]. For large-scale surveillance purposes, a false-negative rate of approximately  20% would not severely affect the identification of houses harboring at least one *T. cruzi*-infected *T. infestans* across several survey occasions. The same argument applies to the detection of house infestation by timed searches [41]. Moreover, the strong household aggregation of triatomine infection implies that the finding of one infected triatomine usually leads to detecting additional infections in the source group (e.g. at the house or village levels). Therefore, stopping examination after finding the first infected bug in a group, or processing individual samples in pools using molecular diagnostics and specifically adapted protocols, would provide key information at reduced cost per unit.

Temperature variations across surveys may be a potential confounder of the associations at issue. Constant temperatures affected the multiplication of *T. cruzi* and differentiation of metacyclic trypomastigotes in *T. infestans*, with a no-growth threshold between 10 °C and 15 °C and a sharp decline over the range 28–37 °C [99]. Despite strong seasonality in the southern Argentine Chaco, in the absence of insecticide spraying domestic populations of *T. infestans* displayed little variations in the monthly prevalence of *T. cruzi* infection between November and April (late spring-early fall), with significantly lower numbers in August (winter) and October (early spring) and peak intensity of infection with epimastigotes and trypomastigotes in November and December [57]. Conversely, in other settings involving intrusive species with extensive sylvatic foci, such as *T. dimidiata* in Yucatan (Mexico), domestic and peridomestic bug infection displayed large seasonal variations [100]. In Pampa del Indio, the cold season is shorter and mean temperatures considerably greater than in the southern Argentine Chaco; 29 of the 37 vector surveys were conducted between October and April [41: Table S1], when average minimum daily temperatures were well above the threshold for parasite multiplication. As triatomines were kept under laboratory conditions between catch and OM-based diagnosis, the time lag provided additional room for developing infections. Consequently, the heterogeneous temporal distribution of triatomine surveys were unlikely to affect the trends in bug infection.

## Conclusions

High-coverage, professional control efforts combined with sustained surveillance-and-response actions based on the judicious application of insecticides gradually or rapidly suppressed the presence and abundance of *T. cruzi*-infected *T. infestans* at a district-wide level depending on the occurrence of pyrethroid resistance. We show that this strategy, conceived within an adaptive management framework, can swiftly diminish vector-borne transmission risks in resource-constrained, remote rural settings of the Gran Chaco. House infestation and bug infection were spatially and temporally aggregated in close association with large socioeconomic inequalities and social vulnerability [42, 50]. Only a small fraction of all housing units ever had an established domestic infestation or bug infection; the baseline entomological data may be used to identify this high-risk group. The low-density infestations with a low prevalence of bug infection recorded after the attack phase suggested minimal transmission risks, and were consistent with the outcomes of serosurveys of *T. cruzi* infection in humans and dogs at endpoint. However, the few surges in domestic bug infection detected postintervention (related to transient domestic recolonization) are a reminder of how easily transmission may reappear in high-risk settings when surveillance becomes more sporadic. More quantitative evidence on the relationship between infected-bug abundance and incidence of human infection is needed to identify eventual transmission thresholds across triatomine species and settings.

## Supplementary Information

**Additional file 1: Figure S1.** Timing of house infestation surveys combined with insecticide spraying by operational area of Pampa del Indio over 2007–2016.

**Additional file 2**: **Text S1.** Checklist of STROBE recommendations for observational studies.

**Additional file 3: Table S1.** Individual triatomine- and house-level data used in current analyses, Pampa del Indio, 2007–2016.

**Additional file 4:** **Figure S2.** Effects of intervention period (baseline, 0 MPI; early, 4–28 MPI, and late, 34–100 MPI) and household ethnicity (Qom relative to Creole households;** A**) or operational area (areas 1–3;** B**) on the marginal predicted mean of bug infection with *Trypanosoma cruzi*. Bars: 95% confidence intervals.

**Additional file 5:** **Figure S3.** Distribution of houses with at least 1 *Triatoma infestans* infected with *Trypanosoma cruzi* according to intervention period with area-wide insecticide spraying in Pampa del Indio, 2007–2016.** A** Baseline (0 MPI),** B** early intervention (4–28 MPI),** C** late intervention (34–100 MPI).

**Additional file 6: Figure S4.** Global spatial analysis of the house-level presence of *Triatoma infestans* infected with *Trypanosoma cruzi* for radial distances of 400, 1000, 2000 and 5000 m (**A**–**D**, respectively) according to intervention period (Baseline, 0 MPI; early, 4–28 MPI; and late, 34–100 MPI) with area-wide insecticide spraying in Pampa del Indio. The observed statistics L(r) are shown as filled circles and the 95% confidence envelopes of the null model as gray lines.

**Additional file 7:** **Figure S5.** Global spatial analysis of the house-level abundance of *Triatoma infestans* infected with *Trypanosoma cruzi* for radial distances of 400, 1000, 2000 Query ID="Q8" Text="References [1, 2, 3, 16, 17, 25 (for which URL should be added): Kindly note changes; modify on proofs if incorrect. Also, provide accessed dates to References [2, 3]." and 5000 m (a–d, respectively) according to intervention period (Baseline, 0 MPI; early, 4–28 MPI; and late, 34–100 MPI) with area-wide insecticide spraying in Pampa del Indio. The observed statistics L(r) are shown as filled circles and the 95% confidence envelopes of the null model as gray lines.

## Data Availability

Information on all relevant data is provided in Additional file 3: Table S1.

## Abbreviations

AUC: Area under the curve
MH: Mantel–Haenszel test
MPI: Months postintervention
OM: Optical microscopy
