# Supplementary figures and images for: The Pampa del Indio project: sustainable vector control and long-term declines in the prevalence and abundance of Triatoma infestans infected with Trypanosoma cruzi in the Argentine Chaco
Source: Parasit Vectors. 2023 Aug 2;16:258. doi: 10.1186/s13071-023-05861-7 (PMC10394798; doi:10.1186/s13071-023-05861-7)

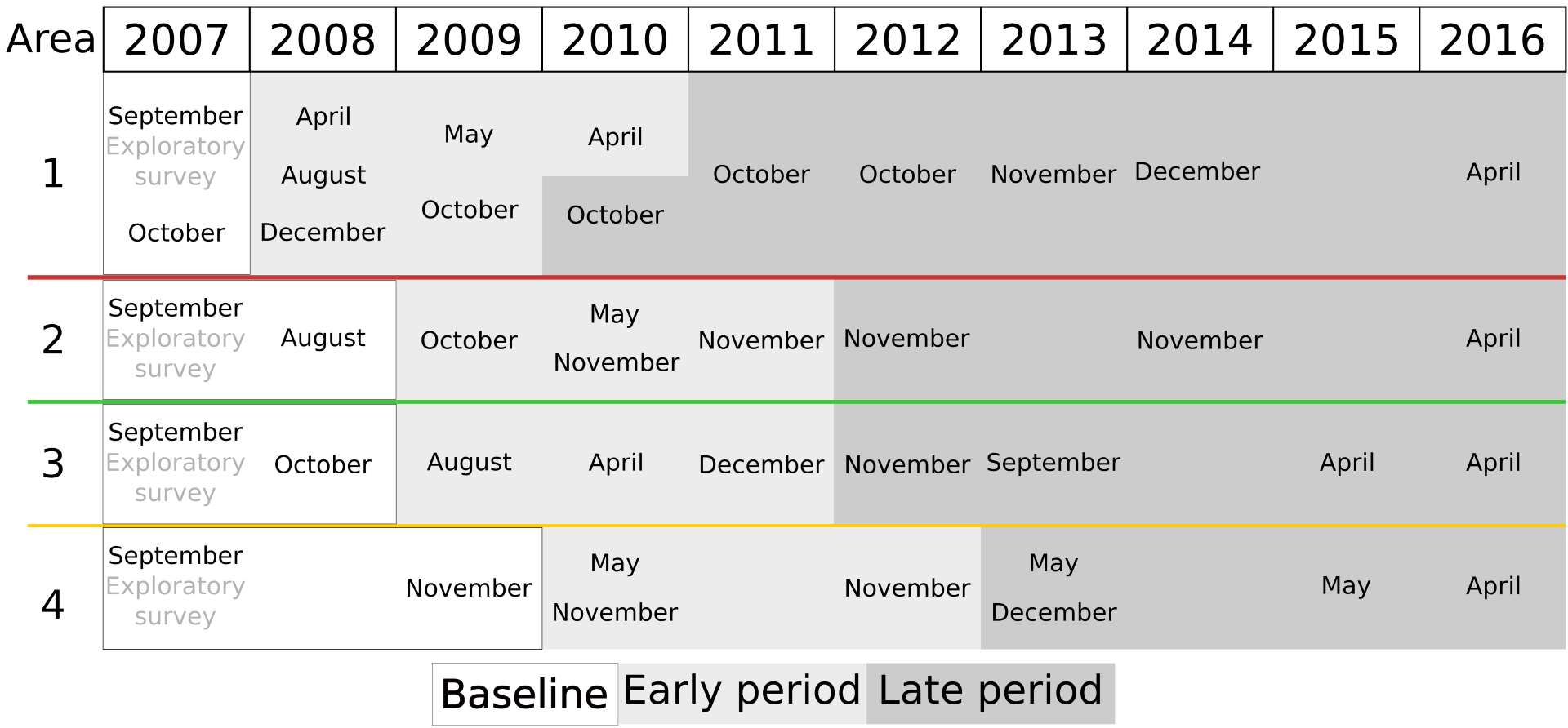

Supplement: Supplementary file 1 — Additional file 1: Figure S1. Timing of house infestation surveys combined with insecticide spraying by operational area of Pampa del Indio over 2007–2016. [file 13071_2023_5861_MOESM1_ESM.tiff]

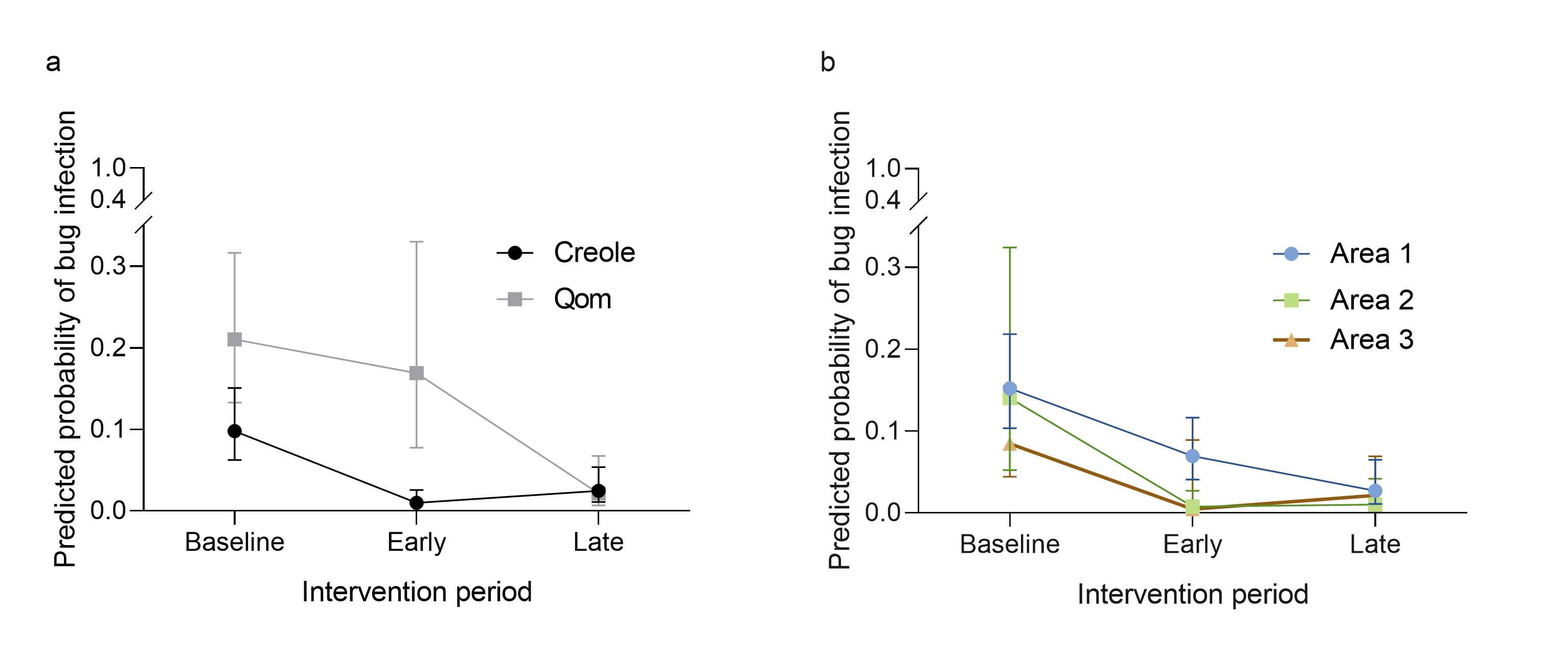

Supplement: Supplementary file 4 — Additional file 4: Figure S2. Effects of intervention period (baseline, 0 MPI; early, 4–28 MPI, and late, 34–100 MPI) and household ethnicity (Qom relative to Creole households; A) or operational area (areas 1–3; B) on the marginal predicted mean of bug infection with Trypanosoma cruzi. Bars: 95% confidence intervals. [file 13071_2023_5861_MOESM4_ESM.tif]

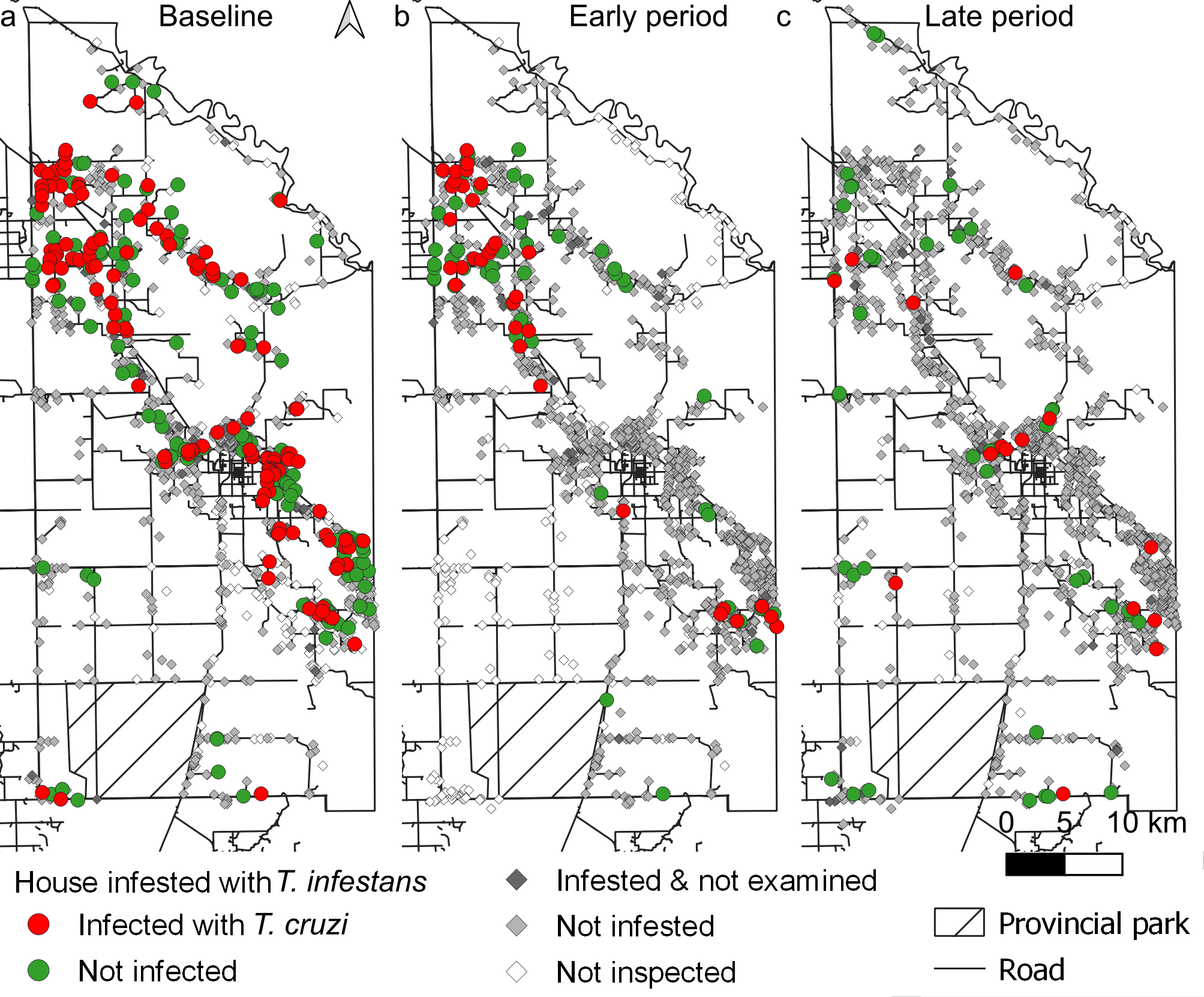

Supplement: Supplementary file 5 — Additional file 5: Figure S3. Distribution of houses with at least 1 Triatoma infestans infected with Trypanosoma cruzi according to intervention period with area-wide insecticide spraying in Pampa del Indio, 2007–2016. A Baseline (0 MPI), B early intervention (4–28 MPI), C late intervention (34–100 MPI). [file 13071_2023_5861_MOESM5_ESM.tiff]

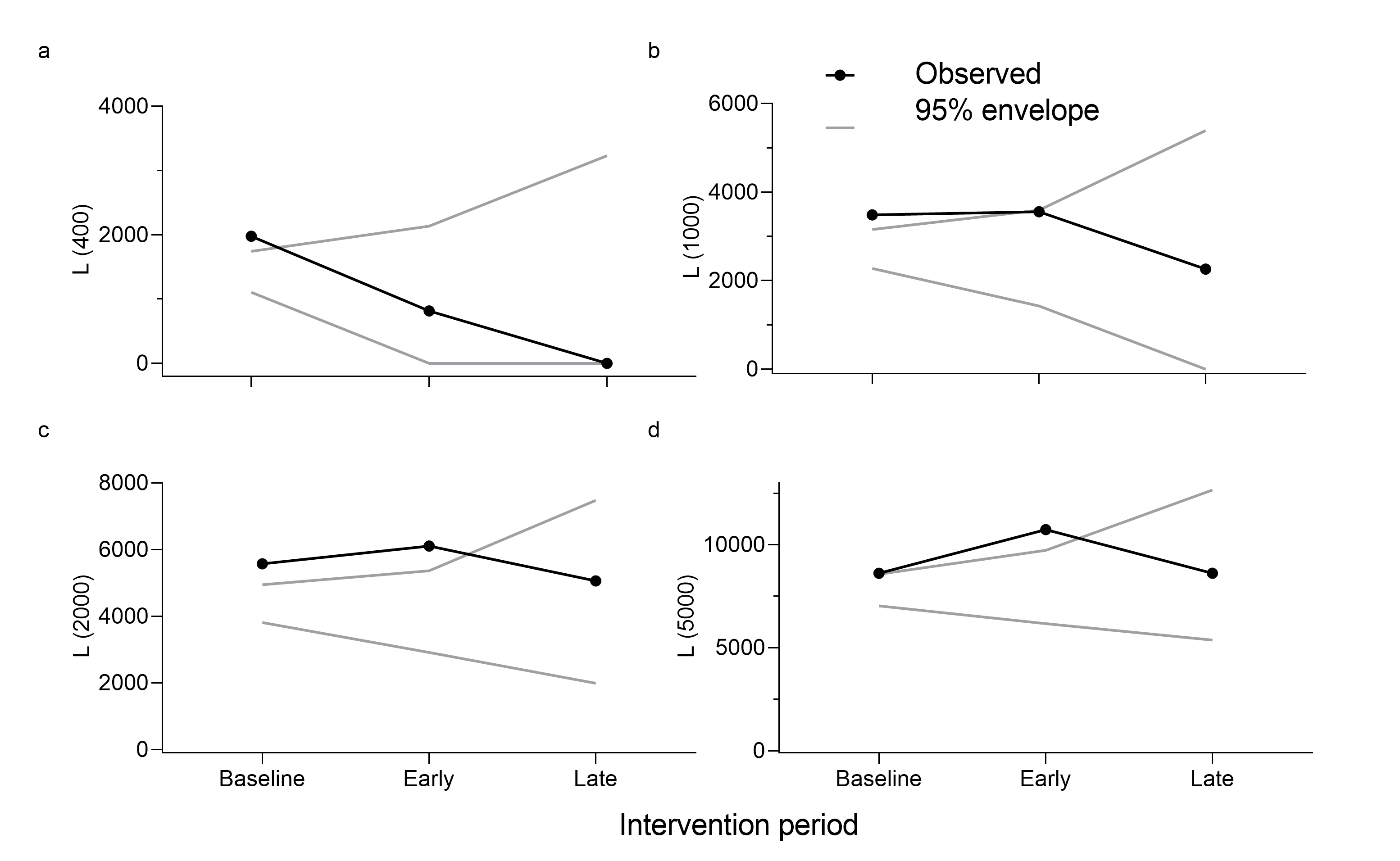

Supplement: Supplementary file 6 — Additional file 6: Figure S4. Global spatial analysis of the house-level presence of Triatoma infestans infected with Trypanosoma cruzi for radial distances of 400, 1000, 2000 and 5000 m (A–D, respectively) according to intervention period (Baseline, 0 MPI; early, 4–28 MPI; and late, 34–100 MPI) with area-wide insecticide spraying in Pampa del Indio. The observed statistics L(r) are shown as filled circles and the 95% confidence envelopes of the null model as gray lines. [file 13071_2023_5861_MOESM6_ESM.tif]

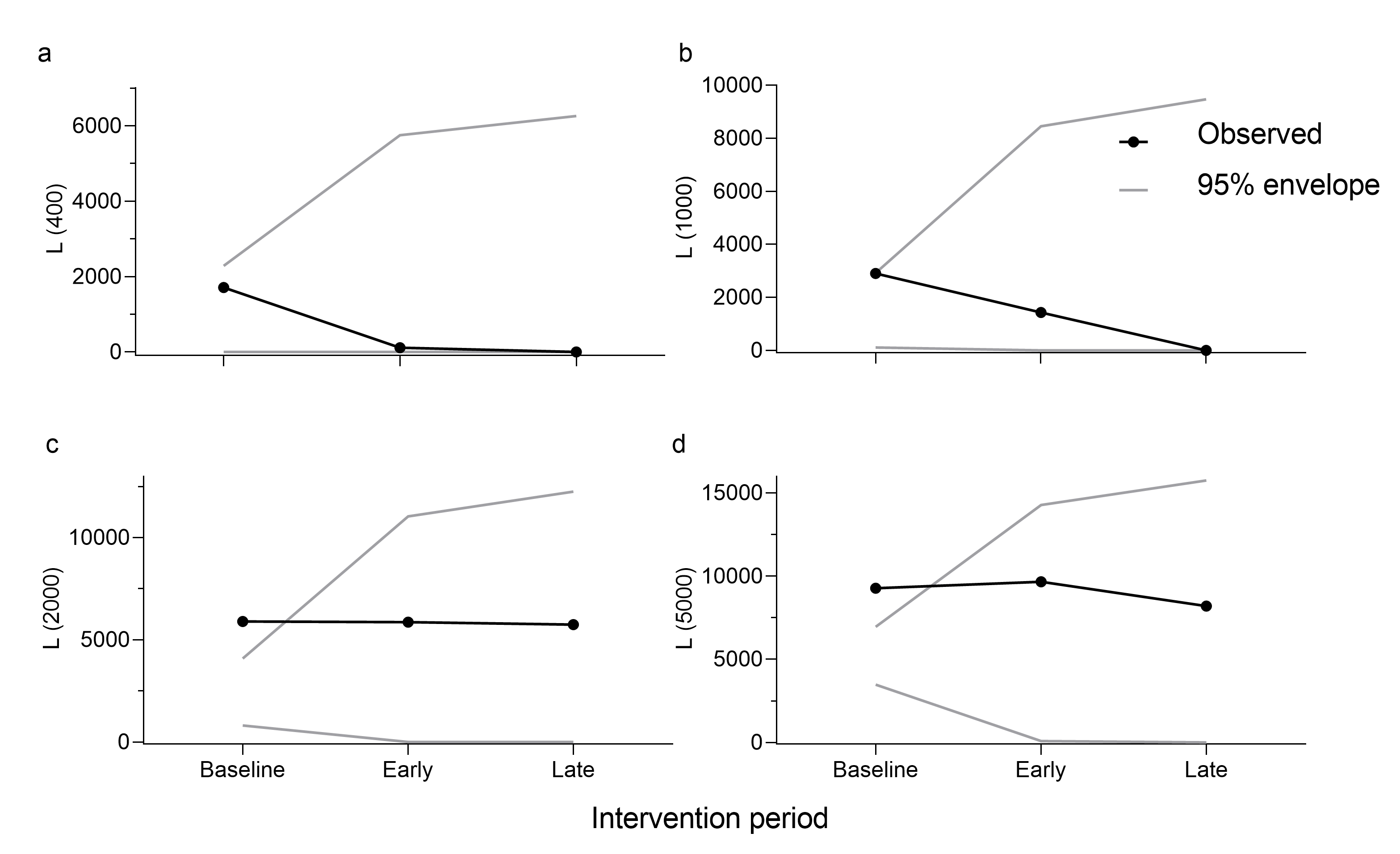

Supplement: Supplementary file 7 — Additional file 7: Figure S5. Global spatial analysis of the house-level abundance of Triatoma infestans infected with Trypanosoma cruzi for radial distances of 400, 1000, 2000 Query ID="Q8" Text="References [1, 2, 3, 16, 17, 25 (for which URL should be added): Kindly note changes; modify on proofs if incorrect. Also, provide accessed dates to References [2, 3]." and 5000 m (a–d, respectively) according to intervention period (Baseline, 0 MPI; early, 4–28 MPI; and late, 34–100 MPI) with area-wide insecticide spraying in Pampa del Indio. The observed statistics L(r) are shown as filled circles and the 95% confidence envelopes of the null model as gray lines. [file 13071_2023_5861_MOESM7_ESM.tif]
