# Supplementary material for: The Pampa del Indio project: sustainable vector control and long-term declines in the prevalence and abundance of Triatoma infestans infected with Trypanosoma cruzi in the Argentine Chaco
Source: Parasit Vectors. 2023 Aug 2;16:258. doi: 10.1186/s13071-023-05861-7 (PMC10394798; doi:10.1186/s13071-023-05861-7)
Supplement: Supplementary file 2 — Additional file 2: Text S1. Checklist of STROBE recommendations for observational studies. [file 13071_2023_5861_MOESM2_ESM.docx]

STROBE Statement—checklist of items that should be included in reports of observational studies

|  | | | Item No. | Recommendation | Page  No. | | | Relevant text from manuscript |
| --- | --- | --- | --- | --- | --- | --- | --- | --- |
| **Title and abstract** | | | 1 | (*a*) Indicate the study’s design with a commonly used term in the title or the abstract | 2 | | | Lines 25- 27 |
|  |  |  |  | (*b*) Provide in the abstract an informative and balanced summary of what was done and what was found | 2 | | | Lines 27-55 |
| Introduction | | | | | | | |  |
| Background/rationale | | | 2 | Explain the scientific background and rationale for the investigation being reported | 3-6 | | | Lines 59-149 |
| Objectives | | | 3 | State specific objectives, including any prespecified hypotheses | 6 | | | Lines 150-176 |
| Methods | | | | | | | |  |
| Study design | | | 4 | Present key elements of study design early in the paper | 7-9 | | | Lines 195-245 |
| Setting | | | 5 | Describe the setting, locations, and relevant dates, including periods of recruitment, exposure, follow-up, and data collection | 7  7-8  8-11 | | | Lines 179-193  Lines 206-235  Lines 242-315  S1 Fig1 |
| Participants | | | 6 | (*a*) *Cohort study*—Give the eligibility criteria, and the sources and methods of selection of participants. Describe methods of follow-up  *Case-control study*—Give the eligibility criteria, and the sources and methods of case ascertainment and control selection. Give the rationale for the choice of cases and controls  *Cross-sectional study*—Give the eligibility criteria, and the sources and methods of selection of participants | 10-11 | | | Lines 286-315 |
|  |  |  |  | (*b*) *Cohort study*—For matched studies, give matching criteria and number of exposed and unexposed  *Case-control study*—For matched studies, give matching criteria and the number of controls per case |  | | |  |
| Variables | | | 7 | Clearly define all outcomes, exposures, predictors, potential confounders, and effect modifiers. Give diagnostic criteria, if applicable | 11-13 | | | Lines 318-340  Lines 341-370  Lines 371-383 |
| Data sources/ measurement | | | 8* | For each variable of interest, give sources of data and details of methods of assessment  (measurement). Describe comparability of assessment methods if there is more than one group | 13 | | | Lines 383-Additional file 3  Table S1 |
| Bias | | | 9 | Describe any efforts to address potential sources of bias | 10  12 | | | Lines 289-294  Lines 349-353 |
| Study size | | | 10 | Explain how the study size was arrived at | 10 | | | Lines 288-289 |
| Quantitative variables | | 11 | | Explain how quantitative variables were handled in the analyses. If applicable, describe which groupings were chosen and why | 12-13 | | Lines 353-372 | |
| Statistical methods | | 12 | | (*a*) Describe all statistical methods, including those used to control for confounding | 11-13 | | Lines 320-385 | |
|  |  |  |  | (*b*) Describe any methods used to examine subgroups and interactions | 12-13 | | Lines 363-368 | |
|  |  |  |  | (*c*) Explain how missing data were addressed | - | |  | |
|  |  |  |  | (*d*) *Cohort study*—If applicable, explain how loss to follow-up was addressed  *Case-control study*—If applicable, explain how matching of cases and controls was addressed  *Cross-sectional study*—If applicable, describe analytical methods taking account of sampling strategy |  | |  | |
|  |  |  |  | (*e*) Describe any sensitivity analyses | 13 | | Lines 369-372 | |
| Results | | | | | | | | |
| Participants | | 13* | | (a) Report numbers of individuals at each stage of study—eg numbers potentially eligible, examined for eligibility, confirmed eligible, included in the study, completing follow-up, and analysed | 13 | | Lines 389-391  Table 1 | |
|  |  |  |  | (b) Give reasons for non-participation at each stage |  | |  | |
|  |  |  |  | (c) Consider use of a flow diagram |  | |  | |
| Descriptive data | | 14* | | (a) Give characteristics of study participants (eg demographic, clinical, social) and information on exposures and potential confounders | 14 | | Lines 397-413  Fig 1 | |
|  |  |  |  | (b) Indicate number of participants with missing data for each variable of interest |  | | Table 1 | |
|  |  |  |  | (c) *Cohort study*—Summarise follow-up time (eg, average and total amount) |  | | Fig 1 | |
| Outcome data | | 15* | | *Cohort study*—Report numbers of outcome events or summary measures over time | 13-21 | | Lines 391-579  Fig 2-7  SFig 1-4 | |
|  |  |  |  | *Case-control study—*Report numbers in each exposure category, or summary measures of exposure |  | |  | |
|  |  |  |  | *Cross-sectional study—*Report numbers of outcome events or summary measures |  | |  | |
| Main results | | 16 | | (*a*) Give unadjusted estimates and, if applicable, confounder-adjusted estimates and their precision (eg, 95% confidence interval). Make clear which confounders were adjusted for and why they were included | 13-14  14  18  18-20  20-21 | | Lines 389-396  Lines 397-413  Tables 1-2  Lines 483-492  Lines 509-552  Fig 4-7  Lines 570 -579 | |
|  |  |  |  | (*b*) Report category boundaries when continuous variables were categorized |  | |  | |
|  |  |  |  | (*c*) If relevant, consider translating estimates of relative risk into absolute risk for a meaningful time period |  | |  | |
| Other analyses | 17 | | Report other analyses done—eg analyses of subgroups and interactions, and sensitivity analyses | | 18-21 | Lines 482-569  Fig 3-4  SFig 2-3 | | |
| Discussion | | | | | | | | |
| Key results | 18 | | Summarise key results with reference to study objectives | | 21 | Lines 582-597 | | |
| Limitations | 19 | | Discuss limitations of the study, taking into account sources of potential bias or imprecision. Discuss both direction and magnitude of any potential bias | | 25-26  22 | Lines 727-746  Lines 610-622 | | |
| Interpretation | 20 | | Give a cautious overall interpretation of results considering objectives, limitations, multiplicity of analyses, results from similar studies, and other relevant evidence | | 21-28 | Lines 582-802 | | |
| Generalisability | 21 | | Discuss the generalisability (external validity) of the study results | | 23 | Lines 647-660 | | |
| Other information | | |  | | | | | |
| Funding | 22 | | Give the source of funding and the role of the funders for the present study and, if applicable, for the original study on which the present article is based | |  | Online subsmission system | | |

*Give information separately for cases and controls in case-control studies and, if applicable, for exposed and unexposed groups in cohort and cross-sectional studies.

**Note:** An Explanation and Elaboration article discusses each checklist item and gives methodological background and published examples of transparent reporting. The STROBE checklist is best used in conjunction with this article (freely available on the Web sites of PLoS Medicine at http://www.plosmedicine.org/, Annals of Internal Medicine at http://www.annals.org/, and Epidemiology at http://www.epidem.com/). Information on the STROBE Initiative is available at www.strobe-statement.org.
